# Permanent Porosity in the Room-Temperature Magnet and Magnonic Material V(TCNE)_2_

**DOI:** 10.1021/acscentsci.3c00053

**Published:** 2023-03-28

**Authors:** Jesse G. Park, David E. Jaramillo, Yueguang Shi, Henry Z. H. Jiang, Huma Yusuf, Hiroyasu Furukawa, Eric D. Bloch, Donley S. Cormode, Joel S. Miller, T. David Harris, Ezekiel Johnston-Halperin, Michael E. Flatté, Jeffrey R. Long

**Affiliations:** † Department of Chemistry, University of California Berkeley, Berkeley, California 94720, United States; ‡ Materials Sciences Division, Lawrence Berkeley National Laboratory, Berkeley, California 94720, United States; § Department of Physics and Astronomy, University of Iowa, Iowa City, Iowa 52242-1479, United States; ∥ Institute for Decarbonization Materials, Berkeley, California 94720, United States; ⊥ Department of Physics, Ohio State University, Columbus, Ohio 43210-1117, United States; ▼ Department of Chemistry, University of Utah, Salt Lake City, Utah 84112-0850, United States; ∇ Department of Applied Physics, Eindhoven University of Technology, Eindhoven 5612 AZ, The Netherlands; ○ Department of Chemical and Biomolecular Engineering, University of California Berkeley, Berkeley, California 94720, United States

## Abstract

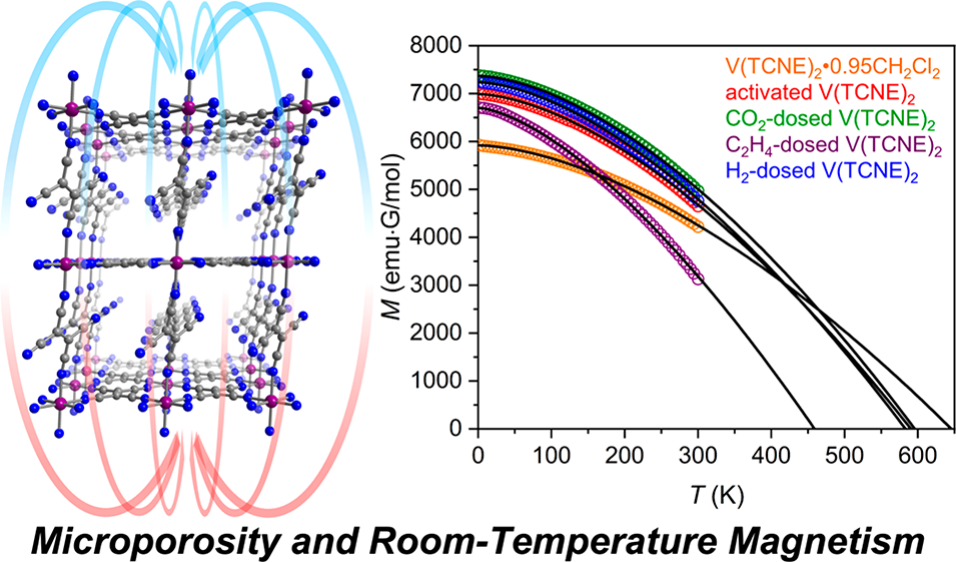

Materials that simultaneously exhibit permanent porosity and high-temperature magnetic order could lead to advances in fundamental physics and numerous emerging technologies. Herein, we show that the archetypal molecule-based magnet and magnonic material V(TCNE)_2_ (TCNE = tetracyanoethylene) can be desolvated to generate a room-temperature microporous magnet. The solution-phase reaction of V(CO)_6_ with TCNE yields V(TCNE)_2_·0.95CH_2_Cl_2_, for which a characteristic temperature of T* = 646 K is estimated from a Bloch fit to variable-temperature magnetization data. Removal of the solvent under reduced pressure affords the activated compound V(TCNE)_2_, which exhibits a T* value of 590 K and permanent microporosity (Langmuir surface area of 850 m^2^/g). The porous structure of V(TCNE)_2_ is accessible to the small gas molecules H_2_, N_2_, O_2_, CO_2_, ethane, and ethylene. While V(TCNE)_2_ exhibits thermally activated electron transfer with O_2_, all the other studied gases engage in physisorption. The T* value of V(TCNE)_2_ is slightly modulated upon adsorption of H_2_ (T* = 583 K) or CO_2_ (T* = 596 K), while it decreases more significantly upon ethylene insertion (T* = 459 K). These results provide an initial demonstration of microporosity in a room-temperature magnet and highlight the possibility of further incorporation of small-molecule guests, potentially even molecular qubits, toward future applications.

## Abstract 2

The molecule-based magnet and magnonic material V(TCNE)_2_ (TCNE = tetracyanoethylene) exhibits magnetic order above room temperature and permanent microporosity and can accommodate incorporation of various gas molecules.

## Introduction

The discovery of materials exhibiting both room-temperature magnetic order and permanent microporosity represents an important scientific challenge, as such materials could enable a wide range of new technologies.^1,2^ For instance, the low-density solid-state structures typically associated with microporous materials could be exploited in the development of new lightweight magnets for implementation in applications such as wind turbines and motor vehicles.^3−5^ Moreover, the presence of regular, atomically precise micropores can allow adsorbate-specific magnetic responses and thus the highly sensitive detection of small-molecule substrates,^6−8^ and these materials may also find use as adsorbents in the separation of paramagnetic gases from diamagnetic gases, such as O_2_ (*S* = 1) from N_2_.^9^ Further, microporous magnets may serve as a chemical platform for fundamental studies of quantum information transfer. In particular, the presence of accessible pores in a magnet that features highly coherent spin wave quasiparticles known as magnons could accommodate paramagnetic molecular guests, potentially enabling the transduction of quantum information via magnon–spin coupling.^10−14^

Despite the potential impact of microporous magnets in fundamental studies and technological applications, no such material has been shown to exhibit an ordering temperature (*T*_c_) above 219 K,^6^ with the vast majority ordering well below 100 K.^1,2,15^ These low ordering temperatures primarily result from the fact that porous magnetic materials generally feature paramagnetic inorganic nodes connected by multiatom diamagnetic linkers, and as such, the paramagnetic centers are coupled via weak superexchange interactions. Since the magnetic ordering temperature is directly correlated to the strength of magnetic coupling between spin centers,^16^ the resulting compounds behave as magnets only at low temperatures.

In pursuit of higher ordering temperatures, many researchers have targeted materials with strong coupling between paramagnetic metal ions and organic radical linkers.^15^ The potential of this approach is exemplified by the amorphous material V(TCNE)_*x*_·*y*CH_2_Cl_2_ (TCNE = tetracyanoethylene; *x* ≈ 2; *y* ≈ 0.5), which was discovered in 1991 to be a bulk ferrimagnet below its thermal decomposition of ∼350 K.^17^ The only other metal–organic magnet known to order above room temperature is the recently discovered crystalline layered two-dimensional material Cr(pz)_2_·0.7LiCl (pz = pyrazine), in which strong magnetic coupling between Cr^II^ and pz^•–^ radical anions gives rise to magnetic order below 515 K.^18^ Of note, studies of dichloromethane-solvated bulk V(TCNE)_*x*_ (*x* ≈ 2)^19,20^ and solvent-free thin films prepared via chemical vapor deposition have reported extrapolated *T*_c_ values as high as 517 and ∼600 K.^21−23^ Recently, thin films of V(TCNE)_*x*_ have also been shown to display coherent magnon transport.^24^

**Figure 1:**
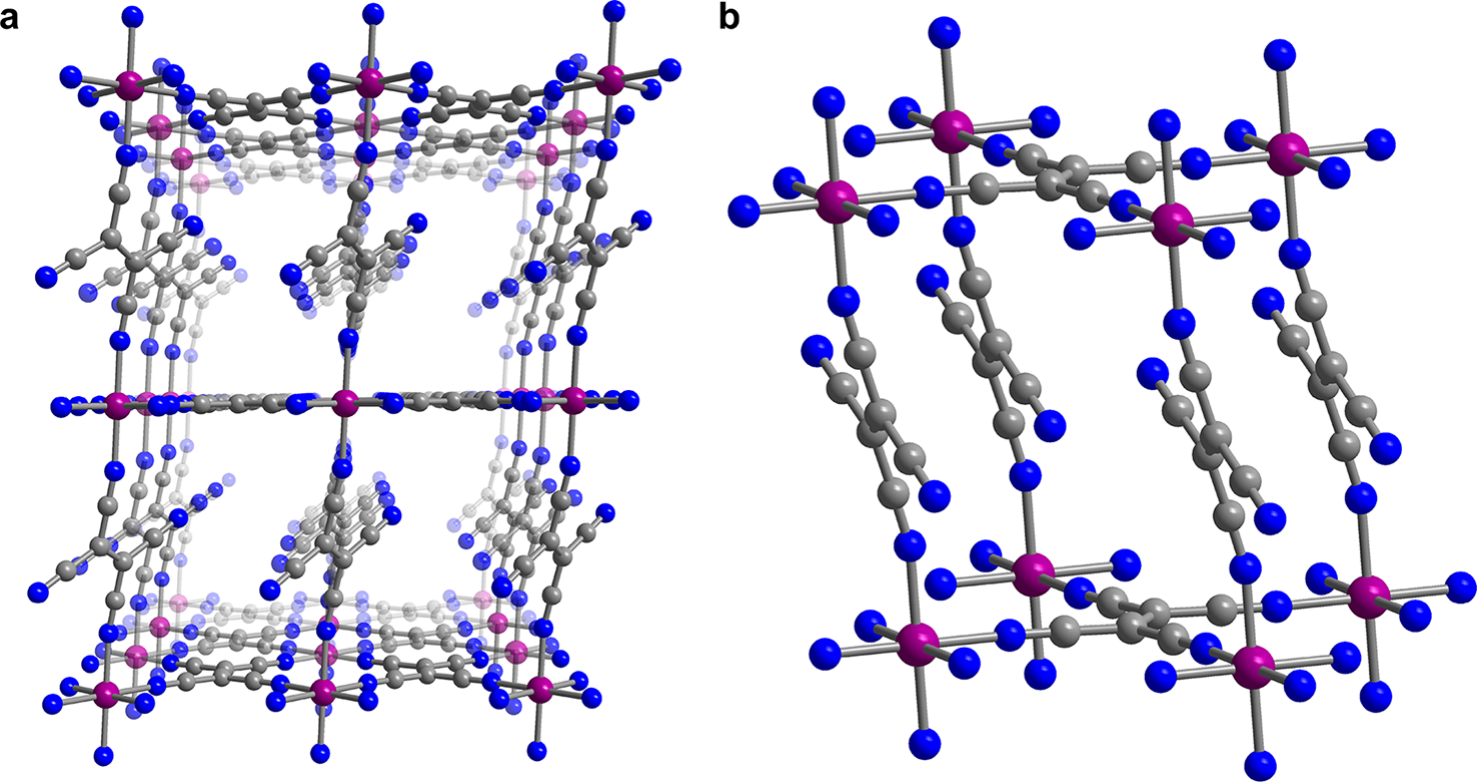
Proposed structure of V(TCNE)_2_ obtained from DFT calculations.^28^ Purple, blue, and gray spheres represent V, N, and C atoms, respectively. (a) Structure as viewed along the crystallographic *b* axis. (b) Expanded view of a portion of the structure, highlighting the presence of potentially accessible voids between sheets of [V(κ^4^-TCNE)]^+^.

Although dozens of studies have been conducted on the chemical and physical properties of variants of V(TCNE)_∼2_·*y*(solvent), a crystalline form has never been reported, and as such, limited knowledge exists regarding its chemical structure.^25^ Spectroscopic investigations support the presence of octahedral V^II^ ions coordinated by a nitrogen atom from six TCNE^•–^ linkers.^25−27^ The high *T*_c_ has been shown to originate from π donation from the diffuse V^II^-based 3d to TCNE^•–^-based π* orbitals.^25,27^ Nevertheless, DFT calculations, taking into account the aforementioned experimental observations, have predicted an open framework structure for V(TCNE)_2_, comprising two-dimensional sheets of [V(κ^4^-TCNE)]^+^ linked by κ^2^-TCNE^–^ pillars (Figure 1), with distorted rectangular openings of 7.2 × 9.9 Å.^28^ This calculated structure, in conjunction with a void volume of 40.3% found in the related structurally characterized material Mn(TCNE)_1.5_(I_3_)_0.5_,^29^ suggests the possibility of permanent porosity in V(TCNE)_2_. Herein, we show that V(TCNE)_2_ can indeed be obtained in a highly porous form through activation of the parent material V(TCNE)_2_·0.95CH_2_Cl_2_. Notably, V(TCNE)_2_ can reversibly adsorb various small molecular guests, including H_2_, N_2_, O_2_, and ethylene, suggesting its potential for applications in magnetic sensing and magnon-based quantum transduction.

## Results and Discussion

### Synthesis

The material V(TCNE)_2_·0.95CH_2_Cl_2_ was synthesized using a modified version of a previously reported procedure.^30^ In brief, a yellow dichloromethane solution of freshly prepared V(CO)_6_^31,32^ (see the Experimental Section) was added dropwise to a stirring colorless dichloromethane solution of tetracyanoethylene. Note that V(CO)_6_ is highly sensitive to light, air, and heat, and hence it is imperative to use the freshly prepared compound for the reaction. Over the course of 1 h, V(TCNE)_2_·0.95CH_2_Cl_2_ precipitated from the reaction solution as a dark green powder, and the presence of 0.95 equiv of dichloromethane was ascertained by combustion elemental analysis (see the Experimental Section). Activated V(TCNE)_2_ was obtained by gently heating V(TCNE)_2_·0.95CH_2_Cl_2_ under reduced pressure (<10 μbar) at 30 °C for 20 h. Note that the solvated and activated materials are notoriously unstable and must be handled with care. Indeed, previous studies have reported that the chemical composition and physical properties of solvated V(TCNE)_∼2_ can vary substantially depending on the reactants, synthetic conditions, and subsequent handling of the product.^19,20,32,33^ In addition, these materials have been reported to decompose even when stored under an inert atmosphere at ambient temperature.^17,34^ Accordingly, the materials reported here were stored at −25 °C when not in use, and the preparation of samples for analysis was carried out immediately following removal from cold storage, under conditions described in the Experimental Section and the Supporting Information. All measurements were performed within three weeks of the chemical synthesis.

### Magnetic Properties

**Figure 2:**
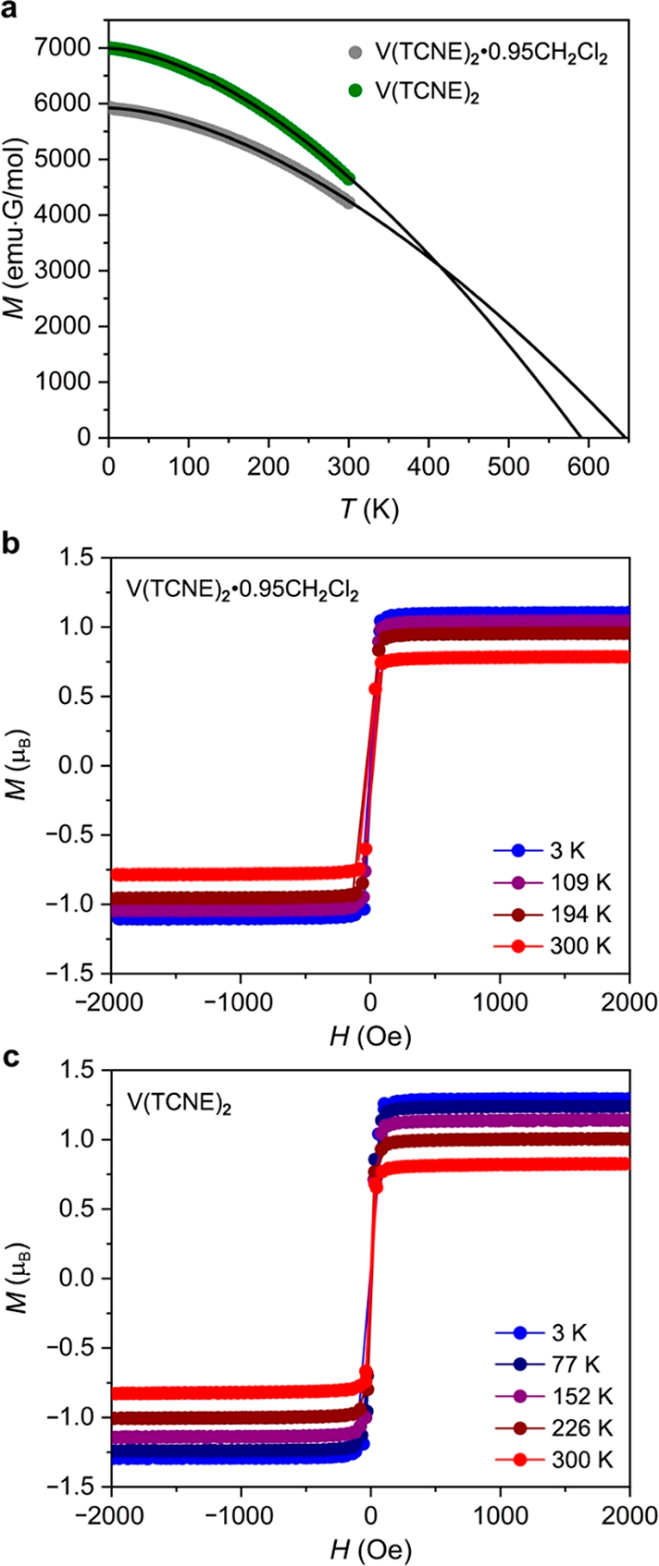
(a) Variable-temperature field-cooled magnetization data for V(TCNE)_2_·0.95CH_2_Cl_2_ (gray) and activated V(TCNE)_2_ (green) collected under a dc field of 0.2 T. Black lines represent fits to the Bloch law. Variable-field magnetization data collected at selected temperatures for V(TCNE)_2_·0.95CH_2_Cl_2_ (b) and activated V(TCNE)_2_ (c).

Variable-temperature dc magnetic susceptibility data were collected for V(TCNE)_2_·0.95CH_2_Cl_2_ over the temperature range 2–300 K to confirm the high magnetic ordering temperature of this solid. A plot of magnetization versus temperature (Figure 2a) reveals that the magnetization monotonically increases upon lowering the temperature from 300 K, indicative of growing magnetic correlation stemming from interactions between *S* = 3/2 V^II^ ions and *S* = 1/2 TCNE^•–^ linkers. While decomposition of the material at ∼350 K precludes the experimental determination of an ordering temperature, the magnetization data were fit to the Bloch law derived from spin wave theory,^22,34−36^*M*(*T*) = *M*(0)(1 – (*T*/T*)^α^), to estimate a characteristic temperature of T* = 646 K (see Section 1.2 of the Supporting Information). Note that T* converges to *T*_c_ in systems where T* is lower than the decomposition temperature. Since the converse is true for V(TCNE)_2_ and a precise determination of *T*_c_ was therefore not possible, T* serves as an indirect probe for characterizing the strength of the exchange interaction. Notably, this is a considerably higher transition temperature than previously reported for dichloromethane-solvated bulk V(TCNE)_*x*_ (*x* ≈ 2; T* as high as 517 K).^19^ The high T* value here may reflect a higher-purity material with fewer defects to arrest magnetic correlation, resulting from the synthetic conditions used and careful handling of the product. Indeed, this value is similar to the value of T* ≈ 600 K estimated for high-quality thin films of V(TCNE)_2_ obtained through chemical vapor deposition,^22,23^ and it represents the highest characteristic temperature yet reported for a bulk coordination solid.^15,18^

A Bloch fit to the resulting magnetization versus temperature data (Figure 2a) for activated V(TCNE)_2_ yielded T* = 590 K, only slightly lower than the temperature of 646 K determined for V(TCNE)_2_·0.95CH_2_Cl_2_. The relatively small decrease of 56 K upon activation is attributed to structural changes resulting from solvent removal—which could originate from the partial collapse of a porous structure—and modified magnetic exchange strength stemming from slight distortions of the vanadium coordination environment.^7,37−40^ Resolvation of the activated V(TCNE)_2_ sample by soaking in dichloromethane for 1 h yielded a material with magnetic properties distinct from that of V(TCNE)_2_·0.95CH_2_Cl_2_, indicating the presence of subtle irreversible changes upon activation (see Figure S1 and Section 1.2 of the Supporting Information).

To probe the nature of the magnetic interaction between the V^II^ ions and TCNE^•–^ linkers further, variable-field magnetization data were collected for V(TCNE)_2_·0.95CH_2_Cl_2_ and V(TCNE)_2_ at selected temperatures between 3 and 300 K (Figure 2b,c). At 3 K, the data for each form of the material exhibit a sharp sigmoidal curve, and the magnetization saturates above 260 Oe at values of 1.1 and 1.3 μ_B_ for the solvated and activated materials, respectively. These values are very close to that of 1.0 μ_B_ expected for antiferromagnetic coupling between one *S* = 3/2 V^II^ ion and two *S* = 1/2 TCNE^•–^ within each formula unit, assuming *g* = 2. Notably, the sharp curves persist up to 300 K, confirming the retention of magnetic order at room temperature. This high-temperature magnetic order results from magnetic exchange between V^II^ and TCNE^•–^, which arises due to the closely matched energies of the radially diffuse dπ and π* orbitals, respectively.^27^ Finally, the lack of significant hysteresis with a small coercivity of *H*_c_ = 40 Oe is consistent with previous reports and is likely due to the amorphous structure and lack of bulk magnetocrystalline anisotropy.^41^

We turned to ferromagnetic resonance (FMR) spectroscopy as an additional probe of the magnetic structure of V(TCNE)_2_·0.95CH_2_Cl_2_ and activated V(TCNE)_2_ at 300 K (Figure S2). Ferromagnetic resonance spectroscopy is a technique analogous to nuclear magnetic resonance or electron paramagnetic resonance spectroscopy, but it operates on magnetic domains in ferromagnetic or ferrimagnetic materials instead of nuclear or electron spins.^42^ Notably, the FMR spectrum for V(TCNE)_2_·0.95CH_2_Cl_2_ features a peak with a resonance field of ∼3550 G, close to that characterized for thin films of V(TCNE)_2_ prepared via chemical vapor deposition (typically ∼3650 G at 9.86 GHz).^22^ Further, this spectrum exhibits a Lorentzian line shape similar to that observed for V(TCNE)_2_ thin films. In contrast, the FMR spectrum for activated V(TCNE)_2_ features a peak at a lower resonance field of ∼3320 G with a long tail to higher fields. The FMR line widths of the solvated and activated samples are both significantly larger than line widths measured for V(TCNE)_2_ thin films (25.5 and 53.5 G versus ∼1.5 G, respectively).^22,23,43^ Relative to V(TCNE)_2_·0.95CH_2_Cl_2_, the greater line width for the activated sample may be explained by the formation of defects and regions of structural inhomogeneity upon desolvation, which would result in a reduction of the magnetic correlation length and create inhomogeneous local resonances, leading to the greater overall peak broadening. Crucially, the presence of a well-resolved peak in the FMR spectrum for activated V(TCNE)_2_ highlights the feasibility of magnon-based transduction involved in the porous form of the material.

### Gas Adsorption in V(TCNE)_2_

**Figure 3:**
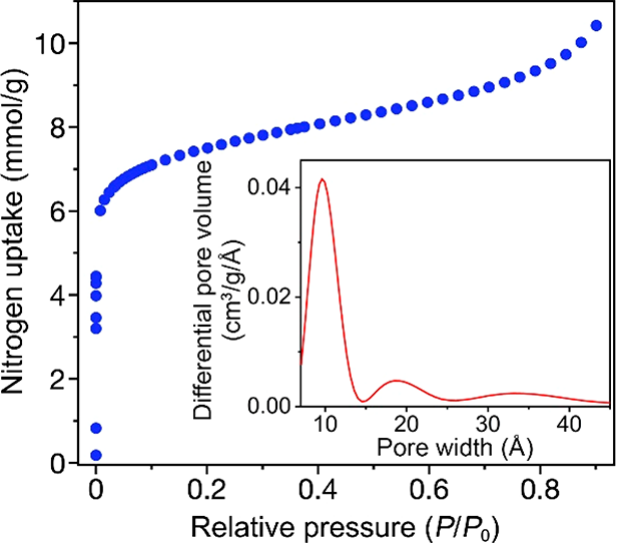
Nitrogen adsorption isotherm obtained for V(TCNE)_2_ at 77 K. Inset: pore size distribution analysis based on Ar adsorption data collected at 87 K (see Figures S4 and S5).

To investigate whether activated V(TCNE)_2_ exhibits permanent porosity, we first collected N_2_ adsorption data at 77 K (Figure 3). Steep N_2_ uptake occurs at low pressures, indicating that the material is indeed permanently microporous, and the data were used to calculate Langmuir and BET surface areas of 850 and 630 m^2^/g, respectively (Figure S3). To the best of our knowledge, V(TCNE)_2_ represents the first example of a porous room-temperature magnet, and it demonstrates the merit in using paramagnetic organic linkers to simultaneously facilitate strong magnetic exchange and give access to porous structures.^15,44^ Importantly, pore size distribution analysis performed based on Ar adsorption data obtained at 87 K (Figure 3, inset; Figures S4 and S5) revealed that V(TCNE)_2_ contains pores with a diameter of ∼9.7 Å, suggesting that insertion of various molecules with smaller kinetic diameters is possible. Indeed, this pore diameter obtained from Ar uptake data is consistent with the calculated structure depicted in Figure 1, which features rectangular pore openings of dimensions 7.2 × 9.9 Å. Moreover, a geometric surface area of 900 m^2^/g was calculated for the aforementioned proposed structure of V(TCNE)_2_, further supporting the presence of microporosity in V(TCNE)_2_ (see Section 1.6 in the Supporting Information).

As an initial test case, we carried out density functional theory (DFT) calculations to probe the feasibility of inserting ethylene into the pores of V(TCNE)_2_ (see Section 1.5 of the Supporting Information), reproducing the aforementioned calculated structure as a starting point (Figure 1).^28^ Two different ethylene loadings, corresponding to V(TCNE)_2_·0.5C_2_H_4_ and V(TCNE)_2_·0.25C_2_H_4_, were modeled by relaxing superstructures of one ethylene molecule within a supercell of two and four unit cells of V(TCNE)_2_, respectively (Figures S6 and S7). By comparing the ground-state energy of the ethylene-loaded systems with that of separated V(TCNE)_2_ and ethylene, we determined formation energies of 0.22 and 0.25 eV for V(TCNE)_2_·0.5C_2_H_4_ and V(TCNE)_2_·0.25C_2_H_4_, respectively. The relatively low formation energies support the feasibility of adsorbing ethylene within V(TCNE)_2_. Further, DFT calculations were performed to assess possible different orientations of the adsorbed ethylene molecules. Relaxations with various starting orientations of ethylene converged to two different final orientations (Figures S6 and S7). For V(TCNE)_2_·0.5C_2_H_4_, phonon calculations indicate modes associated with the rotation of ethylene molecules at phonon frequencies as low as 6.4 meV (52 cm^–1^; Figure S8), suggesting a possible low-energy rotational degree of freedom of the ethylene molecule. Together with the N_2_ adsorption data and pore size analysis, these computational findings provide strong evidence that the porous structure of V(TCNE)_2_ can accommodate small-molecule guests.

**Figure 4:**
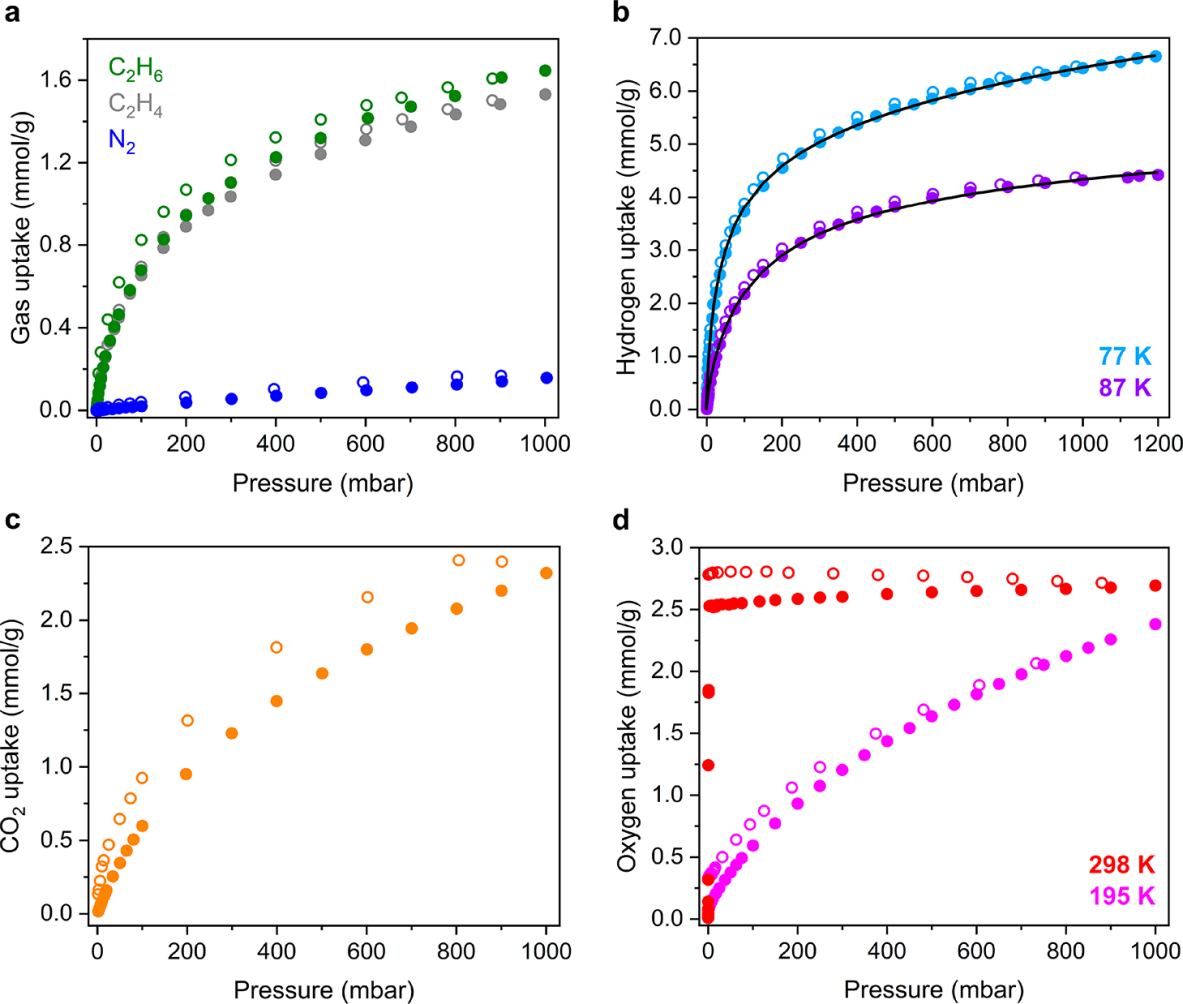
(a) Ethane (green), ethylene (gray), and N_2_ (blue) adsorption (filled circles) and desorption (open circles) isotherms for V(TCNE)_2_ obtained at 298 K (ethane, ethylene) and 291 K (N_2_). (b) Hydrogen adsorption (filled) and desorption (open circles) isotherms for V(TCNE)_2_ obtained at 77 K (light purple) and 87 K (purple). Black lines represent fits using a trisite Langmuir–Freundlich model (see Section 1.4 of the Supporting Information). (c) Carbon dioxide (orange) adsorption (filled circles) and desorption (open circles) isotherms for V(TCNE)_2_ obtained at 291 K. (d) Oxygen adsorption (filled circles) and desorption (open circles) isotherms for V(TCNE)_2_ obtained at 195 K (pink) and 298 K (red).

Based on these computational results, low-pressure ethylene data were collected for V(TCNE)_2_ at 298 K. As shown in Figure 4a, the material indeed adsorbs ethylene at low pressures, reaching a capacity of 1.5 mmol/g at 1.0 bar, corresponding to 0.46 ethylene molecule per formula unit. The isotherm profile suggests moderately strong physisorption of ethylene, whereas stronger ethylene adsorption in materials featuring coordinatively unsaturated metal sites is associated with a steeper initial gas uptake.^45,46^ To further explore the nature of the guest uptake, we collected an ethane isotherm at 298 K for activated V(TCNE)_2_. Ethane uptake in the material is very similar to ethylene uptake, and the data overlay at the lowest pressure points (Figure 4a). At 1.0 bar, the ethane capacity of V(TCNE)_2_ is 1.64 mmol/g (0.50 ethane molecule per formula unit), slightly higher than the ethylene capacity. Interestingly, slight hysteresis is visible in the adsorption/desorption data for both gases, which could indicate some degree of flexibility in the structure of V(TCNE)_2_. Overall, given the similar kinetic diameters and polarizabilities of ethylene and ethane,^47^ these results support physisorption of ethylene in V(TCNE)_2_. However, preliminary results from DFT calculations (Figure S23) and magnetic characterization of ethylene-dosed V(TCNE)_2_ (see below) suggest that the interaction between ethylene and V(TCNE)_2_ may be more complex. Nitrogen uptake in the material at 291 K is very low (Figure 4a), as expected for weak physisorption.^48^

We also examined the H_2_ adsorption properties of V(TCNE)_2_ at cryogenic temperatures (Figure 4b). At 77 K, H_2_ uptake is steep at the lowest pressures and begins to plateau near 4 mmol/g and 125 mbar before reaching a value of 6.65 mmol/g at 1.2 bar (2.04 molecule per formula unit; Figure 4b). Notably, this low-pressure adsorption is steeper than that often observed for H_2_ uptake in materials without exposed metal sites.^49^ At 87 K, H_2_ uptake in V(TCNE)_2_ is less steep, and the material adsorbs 4.42 mmol/g (1.36 molecules per formula unit) at 1.2 bar. The isotherm data at 77 and 87 K were fit using a three-site Langmuir–Freundlich model (Table S1), and the corresponding fit parameters were used with the Clausius–Clapeyron relation to obtain an isosteric heat of adsorption for H_2_ of −7.7 kJ/mol (Figure S9). This value is consistent with moderately strong physisorption and is more exothermic than values associated with H_2_ binding in two well-studied materials for physisorption-based H_2_ storage, Zn_4_O(bdc)_3_ (MOF-5; bdc^2–^ = 1,4-benzodicarboxylate; −4.5 kJ/mol)^49,50^ and the ultraporous framework [Al_3_(μ_3_-O)(H_2_O)_2_(OH)(PET-2)] (NU-1501-Al; PET-2 = expanded triptycene; −4 kJ/mol).^51^ The relatively stronger physisorption may be attributed to the smaller pore size of V(TCNE)_2_ (∼9.7 Å) relative to MOF-5 (12 and 15 Å)^52^ and NU-1501-Al (17 and 22 Å)^51^ and favorable van der Waals interactions between the framework and the H_2_ molecules.

**Figure 5:**
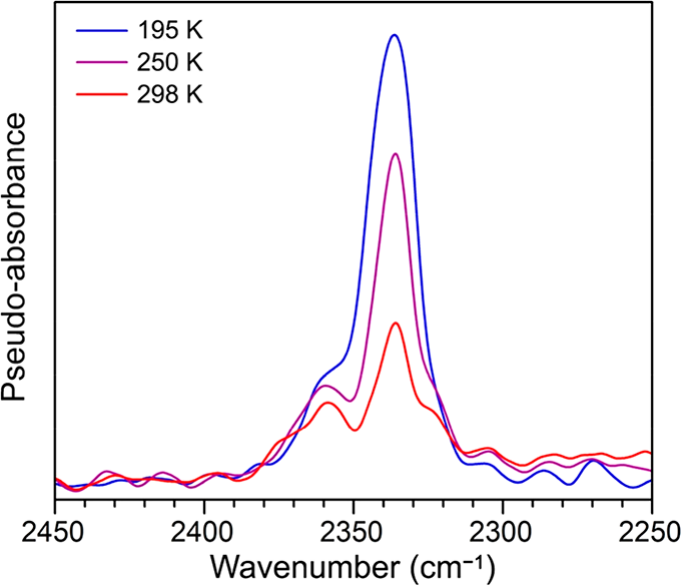
*In situ* DRIFTS spectra for V(TCNE)_2_ dosed with 100 mbar CO_2_ at 195 K (blue), 250 K (purple), and 298 K (red). The *v*_3_ stretch of bound CO_2_ is observed at 2335 cm^–1^, overlapping with peaks corresponding to free CO_2_. A decrease in peak intensity associated with desorption is observed as the temperature is increased.

Low-pressure CO_2_ adsorption data for V(TCNE)_2_ obtained at 291 K reveal gas uptake up to a capacity of 2.32 mmol/g (0.71 molecule per formula unit) at 1.0 bar (Figure 4c), indicative of physisorption. Interestingly, a more pronounced hysteresis is apparent upon CO_2_ desorption than in the case of ethylene or ethane. To further probe interactions between V(TCNE)_2_ and CO_2_, we used *in situ* diffuse reflectance infrared Fourier transform spectroscopy (DRIFTS). Spectra were collected for samples of activated V(TCNE)_2_ dosed with 100 mbar of CO_2_ at 195, 250, and 298 K, and in each case, the v_3_ mode of CO_2_ was observed at 2335 cm^–1^, within the typical range for CO_2_ physisorbed in porous frameworks (Figure 5).^53−55^ In particular, a bathochromic shift of 14 cm^–1^ relative to the gas-phase value of 2349 cm^–1^—rather than the hypsochromic shift typically observed for CO_2_ in cation-exchanged zeolites and metal–organic frameworks with exposed Lewis acidic metal sites—is consistent with physisorption and with the absence of open metal sites in V(TCNE)_2_.

Having established a guest-accessible porous structure for V(TCNE)_2_ and considering the reducing nature of V^II^, we explored the potential of this material to carry out redox-mediated oxygen adsorption. At 195 K, the material adsorbs O_2_ and achieves a capacity of only 2.38 mmol/g (0.73 molecules per formula unit) at 1.0 bar (Figure 4d), behavior indicative of O_2_ physisorption.^56^ Minimal hysteresis is evident upon desorption, although it is slightly more pronounced at the lowest pressures. The small amount of O_2_ remaining in the material may result from strong, irreversible binding at defect sites. At 298 K, O_2_ uptake in V(TCNE)_2_ is initially extremely steep, reaching a value of 2.5 mmol/g at only 3 mbar before plateauing at a value of 2.7 mmol/g (0.83 molecule per formula unit) at 1.0 bar. Desorption data obtained at 298 K revealed more pronounced hysteresis and more O_2_ retained than at 195 K. After being held under dynamic vacuum (<10 μbar) for ∼3 h, the sample adsorbed only minimal O_2_ (Figure S10), indicating irreversible dioxygen binding at this temperature and/or an insufficiently high regeneration temperature. The temperature-dependent O_2_ adsorption data obtained for V(TCNE)_2_ likely originate from a kinetic barrier to thermodynamically favored electron transfer. In such a scenario, insufficient thermal energy would be available at 195 K to overcome this barrier, such that no electron transfer occurs and O_2_ is taken up via physisorption. However, more thermal energy is available at 298 K to induce electron transfer from V(TCNE)_2_ to O_2_. Given the absence of exposed V^II^ sites in the material, O_2_ uptake at 298 K must occur through an outer-sphere electron transfer mechanism, as previously reported for example for the reduced frameworks A_*x*_Fe_2_(bdp)_3_ (A = Na^+^, K^+^; bdp^2–^ = 1,4-benzenedipyrazolate; 0 < *x* ≤ 2).^57^ The latter materials also exhibit steep and irreversible O_2_ uptake at room temperature, although O_2_ uptake becomes partially reversible at higher temperatures.

### Adsorbate-Dependent Magnetic Properties

**Figure 6:**
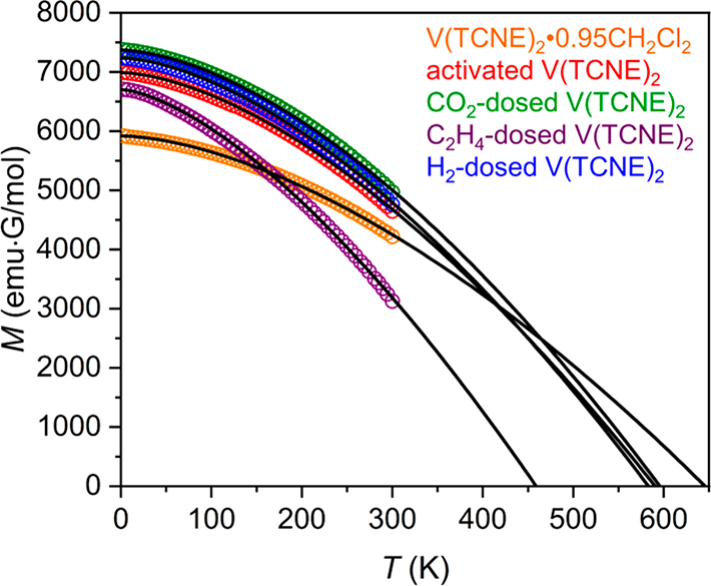
Variable-temperature magnetic susceptibility data collected under a dc field of 2000 Oe for samples of V(TCNE)_2_, before and after dosing with 100 mbar of selected gases. Black lines represent fits to the Bloch law.

Dc magnetic susceptibility data were collected for samples of activated V(TCNE)_2_ after dosing with 100 mbar of CO_2_, H_2_, ethylene, and O_2_ to investigate the impact of these guest molecules on the magnetic properties of the material (see Figure S22 for the results with O_2_). Plots of magnetization versus temperature obtained for activated V(TCNE)_2_ before and after dosing with CO_2_ and H_2_ exhibit similar profiles (Figure 6), and Bloch fits to the data yield T* values of 596 and 583 K for the CO_2_- and H_2_-dosed samples, respectively, close to the value of 590 K estimated for the activated material (see Section 1.2 of the Supporting Information for details). This relatively narrow range of T* values suggests that the physical and electronic structures of the porous V(TCNE)_2_ framework do not significantly change upon uptake of CO_2_ and H_2_, as such changes would very likely lead to larger variations in magnetic exchange and thus the T* value. This scenario is further supported by the similar line widths and resonance fields of the FMR signals of activated V(TCNE)_2_ and samples dosed with CO_2_ and H_2_ (Figure S2).

In contrast, a Bloch law fit to magnetization versus temperature data for a sample of activated V(TCNE)_2_ dosed with 100 mbar of ethylene revealed a much lower characteristic temperature of T* = 459 K, corresponding to a 22% decrease from that of the activated sample. Based on the structures derived from DFT calculations, the insertion of 0.25 equiv of ethylene results in a unit cell expansion by approximately 1.5% in volume, associated with a lengthening of calculated V–N_equatorial_ and V–N_axial_ interatomic distances from 2.06 and 2.06 Å to 2.12 and 2.13 Å, respectively. This structural expansion presumably acts to decrease the spatial overlap of V-based dπ and TCNE^•–^-radical-based π orbitals, in accord with the decreased band dispersions of V-derived spin-up valence states by ∼0.3 eV and the reduced hybridization between V^II^ and TCNE^•–^ (see the Supporting Information and Figure S23). Consequently, this reduced orbital overlap should induce a weakening of the magnetic coupling between spin-bearing V^II^ and TCNE^•–^ units and thus a decrease in T*. In addition to these structural factors, the decrease in T* may also partly stem from a shift of charge density from electron-rich dπ orbitals of V^II^ to the unfilled π* orbitals of the TCNE^•–^ linkers.

## Conclusion

We have synthesized high-quality bulk V(TCNE)_2_·0.95CH_2_Cl_2_ with an estimated characteristic temperature of T* = 646 K, the highest reported to date for any coordination solid. Careful activation of V(TCNE)_2_·0.95CH_2_Cl_2_ yields the permanently porous material V(TCNE)_2_, which behaves as a bulk ferrimagnet above room temperature with an estimated T* = 590 K. Significantly, the activated compound exhibits a rigid pore structure with an empirically derived average pore diameter of ∼9.7 Å and reversibly adsorbs H_2_, CO_2_, and ethylene via physisorption. At 195 K, the material also reversibly physisorbs O_2_. While the presence of H_2_ or CO_2_ guest molecules does not significantly impact the magnetic properties of V(TCNE)_2_, introduction of ethylene into the porous structure results in a significant decrease in characteristic temperature to T* = 459 K, likely reflecting stronger interactions with the TCNE^•–^ radical linkers of the material. Future work will entail the incorporation of paramagnetic guest molecules, including small molecules that can act as qubits, and subsequent studies of magnon–spin quantum transduction.

## Experimental Section

### General Considerations

Unless otherwise noted, all manipulations were carried out under an argon atmosphere in an Mbraun MB200MOD glovebox. Glassware was dried in an oven at 150 °C for at least 12 h and allowed to cool in an evacuated glovebox antechamber prior to use. Dichloromethane was dried using a commercial solvent purification system made by JC Meyer Solvent Systems and stored over 3 Å molecular sieves prior to use. Tetracyanoethylene (96% purity) was purchased from Sigma-Aldrich, purified through vacuum sublimation at 140 °C and 40 μbar, and subsequently stored under an argon atmosphere. The compounds (Et_4_N)[V(CO)_6_] and V(CO)_6_ were prepared according to the previously reported procedures.^22,31^ Carbon, hydrogen, and nitrogen elemental analyses were obtained from the Microanalytical Laboratory at the University of California Berkeley.

### Safety Notes

Vandium hexacarbonyl is highly toxic and sensitive to air, light, and temperature. It must be handled under inert gas and used immediately following synthesis for subsequent manipulations. In addition, tetracyanoethylene hydrolyzes in moist air to yield toxic hydrogen cyanide. It must be stored dry, and any waste must be stored in a solution with basic pH.

### Synthesis of V(TCNE)_2_·0.95CH_2_Cl_2_

The synthesis of V(TCNE)_2_·0.95CH_2_Cl_2_ was carried out using a modification of a previously reported procedure.^30^ A yellow solution of V(CO)_6_ (75.0 mg, 0.342 mmol) in 3 mL of dichloromethane was added dropwise in a 20 mL glass scintillation vial containing a colorless solution of TCNE (87.6 mg, 0.684 mmol) in 12 mL of dichloromethane. The dark blue-green suspension was stirred for 10 min until the evolution of CO gas ceased. The vial was sealed with a polytetrafluoroethylene-lined cap, and the reaction mixture was stirred at 25 °C for 1 h. The resulting suspension was filtered with a Nylon membrane filter, washed with CH_2_Cl_2_ (4 × 2 mL), and dried to yield 127 mg (96%) of product as a dark green powder. Two batches of samples were prepared using an identical synthetic procedure. Note that the chemical and physical properties of solvated V(TCNE)_*x*_ (*x* ≈ 2) are known to vary across different batches of samples depending on the purity of the reactants, the synthetic conditions, and subsequent handling of the material.^19,20,32,33^ Anal. Calcd for VC_12.95_H_1.9_N_8_Cl_1.9_ (%): C, 40.11; H, 0.49; N, 28.89. Found (%): C, 39.85; H, 0.41; N, 29.14.

### Activation of V(TCNE)_2_·0.95CH_2_Cl_2_

The compound V(TCNE)_2_·0.95CH_2_Cl_2_ (70.0 mg) was transferred to a preweighed analysis tube. The tube was capped with a Micromeritics TranSeal and evacuated by heating at 30 °C under dynamic vacuum (<10 μbar) for 20 h when an outgas rate of less than 3 μbar/min was recorded. The sample was then transferred to a solvent-free N_2_ glovebox to yield 51.2 g (92%) of product as a dark green powder, which was immediately transferred for storage at −25 °C. Anal. Calcd for VC_12.06_H_0.12_N_8_Cl_0.12_ (%): C, 46.39; H, 0.04; N, 35.89. Found (%): C, 46.43; H, 0.12; N, 35.88.

### Gas Adsorption Measurements

Gas adsorption isotherms were collected between 0 and 1 bar using a volumetric method with a Micromeritics ASAP 2020 instrument. In an N_2_-filled glovebox, a typical sample of 30–50 mg was transferred to a preweighed analysis tube, which was capped with a Micromeritics TranSeal and evacuated by heating at 30 °C with a ramp rate of 0.2 °C/min under dynamic vacuum for 20 h. The evacuated analysis tube containing the degassed sample was then carefully transferred to an electronic balance and weighed again to determine the mass of sample. The tube was then transferred back to the analysis port of the gas adsorption instrument. The outgas rate was again confirmed to be less than 3 μbar/min. For all isotherms, warm and cold free-space correction measurements were performed using ultrahigh-purity He gas (UHP grade 5.0, 99.999% purity). Oil-free vacuum pumps and oil-free pressure regulators were used for all measurements to prevent contamination of the samples during the evacuation process or of the feed gases during the isotherm measurements. Pore-size distributions were calculated using the DFT method with a quenched solid-state DFT (QSDFT) model of Ar at 87 K adsorbed in carbon with cylindrical pores, as implemented in the Quantachrome *VersaWin* software.

### Infrared Spectroscopy

Infrared spectra were collected using a Bruker Vertex 70 spectrometer equipped with a glowbar source, KBr beam splitter, and a liquid-nitrogen-cooled mercury–cadmium–telluride detector. A custom-built diffuse reflectance system with an IR-accessible gas-dosing cell was used for all measurements. The sample temperature was controlled by an Oxford Instruments OptistatDry TLEX cryostat, and the sample atmosphere was controlled by a Micromeritics ASAP 2020Plus gas adsorption analyzer. A sample of desolvated V(TCNE)_2_ was dispersed in dry diamond powder (45 μm, 10 wt % framework) by gently mixing the two powders in a N_2_-filled glovebox. After cooling to 195 K, 100 mbar of CO_2_ was dosed into the cell, which was then sealed. Spectra were collected continuously until no changes were observed. Collection was repeated at 250 and 298 K.

### Magnetic Measurements

Samples of V(TCNE)_2_ were prepared by adding powder to a 5 mm i.d. × 7 mm o.d. quartz tube containing a raised quartz platform. These samples included solvated V(TCNE)_2_·0.95CH_2_Cl_2_ (11.8 mg), activated V(TCNE)_2_ (11.2 mg), activated V(TCNE)_2_ dosed with CO_2_ (11.0 mg), activated V(TCNE)_2_ dosed with H_2_ (14.9 mg), activated V(TCNE)_2_ dosed with C_2_H_2_ (12.0 mg), and activated V(TCNE)_2_ dosed with O_2_ (10.4 mg). Powder samples were restrained with a plug of compacted glass wool that prevented crystallite torqueing but enabled gas dosing. The gas-dosed samples were prepared by attaching quartz tubes containing activated V(TCNE)_2_ to a Micromeritics ASAP 2020 HD gas adsorption analyzer and dosing with CO_2_, H_2_, or ethylene to 100 mbar and O_2_ to 150 mbar at 25 °C (see Figure S22 for magnetic data collected for the O_2_-dosed sample). A sample of activated V(TCNE)_2_ resolvated with CH_2_Cl_2_ (14.4 mg) was also prepared as follows: inside an argon glovebox, activated V(TCNE)_2_ was soaked in dichloromethane solution for 1 h, filtered on a polypropylene membrane filter (0.45 μm), and dried briefly at ambient temperature under reduced pressure before being loaded into a quartz tube. After sample preparation, all quartz tubes were flame-sealed while the sample was cooled with liquid nitrogen. All magnetic measurements were performed using a Quantum Design MPMS2 SQUID magnetometer from 3 to 300 K at applied magnetic fields ranging from 0 to ±7 T. ac susceptibility measurements were performed with an oscillating field of 4 Oe with a frequency from 10 to 100 Hz (see the Supporting Information and Figures S13–S15 and S19–S21). Diamagnetic corrections were applied to the data using Pascal’s constants of χ_D_ = −0.00019390 emu mol^–1^ for the solvated samples and χ_D_ = −0.00015356 emu mol^–1^ for the activated and gas-dosed samples.

### Ferromagnetic Resonance Spectroscopy

A typical sample of ∼30 mg of material was transferred to a 25 cm long quartz tube with an outer diameter of 4 mm. The gas-dosed samples were prepared by attaching the quartz tubes containing activated V(TCNE)_2_ to a Micromeritics ASAP 2020 gas adsorption analyzer and then dosing with CO_2_ and H_2_ at 25 °C. All quartz tubes containing samples were flame-sealed while the sample was cooled with liquid nitrogen under a static gas atmosphere. Ambient-temperature FMR spectroscopy was carried out using a Bruker EPR spectrometer configured with an X-band bridge providing 200 μW of applied microwave power and a modulation field and frequency of 0.03 G and 100 kHz, respectively. Under standard operation, the microwave frequency is tuned between 9 and 10 GHz for optimal microwave cavity performance before the measurement, and then the frequency is fixed while the dc field is swept during the measurement.

### Electronic Conductivity Measurements

A powder sample of ∼10 mg of V(TCNE)_2_·0.95CH_2_Cl_2_, activated V(TCNE)_2_, or “aged” V(TCNE)_2_ (stored under Ar at 295 K for 1 month) was pressed into a pellet with a custom-built copper screw cell (Figure S12). Electronic conductivity values (Table S2) were determined through both *I*–*V* measurements and electrochemical impedance spectroscopy using a Bio-Logic VMP-3 multipotentiostat fitted into an argon atmosphere glovebox. Data were analyzed with the EC-Lab v10.41 software package from Bio-Logic. *I*–*V* profiles were collected for a ±1 V voltage window and fitted with Ohm’s law, *V* = *I·R*, where *V* is the voltage, *I* is the current, and *R* is the resistance. The resistance and volume of each pressed pellet sample were used to calculate the conductivity, σ, using the equation σ = *L*/(*R·A*), where *L* and *A* are the thickness and contact area of a cylindrical pellet, respectively. For impedance measurements, unless otherwise noted, all data were collected at 250 mV ac and 0 V dc bias, with a frequency sweep range of 1 MHz to 1 Hz with sampling of 15 points per decade, averaging 10 measurements per frequency. Data were fitted to a model circuit. Similar electronic conductivity values of ∼1 × 10^–3^ S cm^–1^ were measured for V(TCNE)_2_ and V(TCNE)_2_·0.95CH_2_Cl_2_ (see Table S2 and Figures S16 and S17), indicating that the material is stable to activation, and these values are consistent with previous electronic conductivity values reported for V(TCNE)_*x*_·*y*CH_2_Cl_2_ (*x* ≈ 2; *y* ≈ 0.5).^58,59^ In contrast, a decreased electronic conductivity of ∼9 × 10^–5^ S cm^–1^ was measured for V(TCNE)_2_ after it had been stored under Ar atmosphere at 295 K for 1 month (see Table S2 and Figure S18).

## Supporting Information Available

Additional experimental procedures and discussions, gas adsorption data, ferromagnetic resonance data, additional magnetic data, electronic conductivity data, and additional computational details (PDF)
