# Supplementary material for: Permanent Porosity in the Room-Temperature Magnet and Magnonic Material V(TCNE)2
Source: ACS Cent Sci. 2023 Mar 28;9(4):777–86. doi: 10.1021/acscentsci.3c00053 (PMC10141614; doi:10.1021/acscentsci.3c00053)
Supplement: Supplementary file 1 — oc3c00053_si_001.pdf [file oc3c00053_si_001.pdf]

Supporting Information for:

Permanent Porosity in the Room-Temperature Magnet and Magnonic Material $V(TCNE)_2$

Jesse G. Park,[a,‡] David E. Jaramillo,[a,b,‡] Yueguang Shi,[c] Henry Z. H. Jiang,[a,b,d] Huma Yusuf,[e] Hiroyasu Furukawa,[a,b,d] Eric D. Bloch,[a] Donley S. Cormode,[e] Joel S. Miller,[f] T. David Harris,[a,d] Ezekiel Johnston-Halperin,[e] Michael E. Flatté,[c,g] Jeffrey R. Long[a,b,d,h,]*

[a] *Department of Chemistry, University of California Berkeley, Berkeley, California 94720, United States*
[b] *Materials Sciences Division, Lawrence Berkeley National Laboratory, Berkeley, California 94720, United States*
[c] *Department of Physics and Astronomy, University of Iowa, Iowa City, Iowa 52242-1479, United States*
[d] *Institute for Decarbonization Materials, Berkeley, California 94720, United States*
[e] *Department of Physics, Ohio State University, Columbus, Ohio 43210-1117, United States*
[f] *Department of Chemistry, University of Utah, Salt Lake City, Utah 84112-0850, United States*
[g] *Department of Applied Physics, Eindhoven University of Technology, Eindhoven 5612 AZ, The Netherlands*
[h] *Department of Chemical and Biomolecular Engineering, University of California Berkeley, Berkeley, California 94720, United States*

*Correspondence email: jrlong@berkeley.edu (J.R.L.)

Table of Contents

1. Additional experimental methods 2
1.1 Synthesis 2
1.2 Additional magnetic characterization data 2
1.3 Ferromagnetic resonance spectroscopy 3
1.4 Gas adsorption isotherm fitting 3
1.5 Computational details 4
1.6 Surface area calculations 4
2. Supporting Tables 6
3. Supporting Figures 7
4. References 23

1. Additional experimental methods

1.1 Synthesis

Previous reports suggest that the chemical compositions and physical properties of solvated V(TCNE)$_x$ ($x \approx 2$) may vary depending on the starting materials, synthetic conditions, and subsequent handling.[1-4] To check for the consistency of different batches of samples, two batches of the V(TCNE)$_2$·0.95CH$_2$Cl$_2$ and activated V(TCNE)$_2$ were obtained using identical synthetic procedures. First, ethylene adsorption isotherms (Figure S11) exhibit similar gas uptake for the two batches, suggesting similar pore environments and accessible surface areas. A Bloch fit to the variable-temperature dc magnetic susceptibility data for the first batch of V(TCNE)$_2$·0.95CH$_2$Cl$_2$ yielded an estimated magnetic ordering temperature of 640 K, which is quite close to 646 K for the second batch. This result confirms a small variability between the two batches obtained using an identical synthetic procedure. Further, upon activation of the two V(TCNE)$_2$·0.95CH$_2$Cl$_2$ batches, a similar decrease of the estimated ordering temperature was observed (from 640 to 570 and 646 to 590 K, respectively). Slight difference in the magnetic ordering temperatures may be due partial collapse of the porous structure of the activated phase, and further investigation is needed. For the magnetic characterization of the first batch, 13.8 and 15.8 mg of V(TCNE)$_2$·0.95CH$_2$Cl$_2$ and activated V(TCNE)$_2$, respectively, were used.

1.2 Additional magnetic characterization data

Ac magnetic susceptibility data collected for solvated V(TCNE)$_2$·0.95CH$_2$Cl$_2$ (Figure S13) feature broad, frequency-dependent in-phase (χ_M') and out-of-phase (χ_M'') susceptibility peaks, consistent with previously reported glassy magnetism.[2] Upon activation, V(TCNE)$_2$ exhibits a similar ac magnetic susceptibility profile, suggesting that glassy magnetism is retained. Variable-field magnetization data collected for activated V(TCNE)$_2$ at 3 K (see Figure S14) reveal a magnetization value that saturates at 1.3 μ_B/mol. This value is slightly higher than the saturation magnetization of 1.1 μ_B/mol observed for V(TCNE)$_2$·0.95CH$_2$Cl$_2$, and the increase is most likely due to increased g from modified coordination environments around metal centers following slight pore collapse and structural reorganization. To investigate the reversibility of the magnetic properties, magnetic susceptibility data was collected for V(TCNE)$_2$ resolvated with dichloromethane (Figure S15). Assuming that the resolvated sample should have similar magnetic structure and saturation magnetization as the activated V(TCNE)$_2$, dichloromethane content of ~0.35 equivalents per formula unit was deduced for the resolvated sample. Notably, this is significantly smaller than observed for the solvated V(TCNE)$_2$·0.95CH$_2$Cl$_2$. This observation suggests the smaller pore volume for the activated and resolvated V(TCNE)$_2$ compared to V(TCNE)$_2$·0.95CH$_2$Cl$_2$ and that the resolvation doesn't return the magnetic properties, most likely due to the irreversibility of the structural reorganization. At 3 K, activated samples of V(TCNE)$_2$ dosed with CO_2 and H_2 exhibit similar saturation magnetization values of ~1.3 μ_B/mol (see Figures S19 and S20). While dosing activated V(TCNE)$_2$ with O_2 at 300 K results in a non-magnetic phase (Figure S22) due to outer-sphere electron transfer to O_2, adsorption data collected at 195 K suggest that this process is hindered at lower temperatures. The magnetic properties of V(TCNE)$_2$ dosed with O_2 at lower temperatures is under investigation.

To a first order approximation, temperature dependent magnetization may be fitted using the following Bloch's law.

$M(T) = M(0)(1-(T/T^*)^{3/2})$

where $M(T)$ is saturation magnetization, $M(0)$ is saturation magnetization at 0 K, T is temperature, and T^* is characteristic temperature. $M(0)$ was assigned as the saturation magnetization at the lowest experimentally accessible temperature of 3 K. While typical bulk magnets follow the Bloch's $T^{3/2}$ law, glassy magnets and magnetic nanoparticles often exhibit deviation from this temperature dependence. As such, Bloch's law may be modified as following.

$M(T) = M(0)(1-(T/T^*)^{\alpha})$

For all $V(TCNE)_2$ samples, the modified Bloch equation was used to fit the dc magnetic susceptibility data. Excellent fits ($R^2 > 0.99$) were obtained for all samples with α of ~1.6, slight deviations from the Bloch's $T^{3/2}$ law as expected. As the fits represent best lines of fit obtained by minimizing the residual sum of error squares, T^*'s do not carry error bars.

1.3 Ferromagnetic resonance spectroscopy

Ferromagnetic resonance (FMR) spectra were collected for bulk powder samples of $V(TCNE)_2{\cdot}0.95CH_2Cl_2$, activated $V(TCNE)_2$, and gas-dosed $V(TCNE)_2$ at 300 K. As discussed in the main text, the FMR spectrum of the activated sample exhibits a larger linewidth and smaller resonance field than that of the solvated sample (Figure S2). In addition to the reasons highlighted in the main text, another possible reason for these observed differences could be due to local anisotropy in the g-factor in the activated sample relative to the solvated sample. Increased anisotropy in the g-factor could arise from dipole-dipole interactions (van der Waals coupling) between adjacent ligands and metal atoms resulting from partial pore collapse; this could result in local pockets of randomly oriented magnetic spins, which would increase the rate at which resonant spins dephase, causing higher overall damping or linewidth broadening. It should also be noted that the FMR spectra of gas-dosed samples of activated $V(TCNE)_2$ exhibit slight shifts in their resonance fields relative to that determined for the activated sample (shifts of up to 30 G). Although the saturation magnetization is expected to be the same for the activated and gas-dosed samples, slight differences in the g-factor anisotropy of the gas-dosed samples relative to the activated sample can cause a net change in the local and bulk magnetization orientation, and hence the saturation magnetization.

1.4 Gas adsorption isotherm fitting

Low-pressure isotherms were fit with a tri-site Langmuir Freundlich equation (eq. 1, see Table S1), where n is the total amount of adsorbed gas in mmol/g, P is the pressure in bars, $n_{\mathrm{sat},i}$ is the saturation capacity in mmol/g, b_i is the Langmuir parameter in bar^{-1} defined in eq. 2, and v_i is the Freundlich parameter for each site.

$$n = \frac{n_{sat,1} b_1 P^{v_1}}{1+b_1 P^{v_1}} + \frac{n_{sat,2} b_2 P^{v_2}}{1+b_2 P^{v_2}} + \frac{n_{sat,3} b_3 P^{v_3}}{1+b_3 P^{v_3}} \quad (1)$$

$$b_i = e^{S_i/R} e^{\frac{-E_i \times 1000}{RT}} \quad (2)$$

For eq. 2, S_i is the site-specific entropy of adsorption in units of J/mol/K, E_i is the enthalpy of adsorption in units of kJ/mol, R is the ideal gas constant in units of J/mol/K, and T is the temperature in K.

1.5 Computational details

Density functional theory (DFT) calculations were carried out using the VASP 5.4.4 package.[5-7] The VASP DFT algorithm uses a plane-wave basis set and pseudopotentials for the electron potentials. All the pseudopotentials used in our DFT calculations were from the VASP official projector augmented wave (PAW) pseudopotential set, with five valence electrons per vanadium, four valence electrons per carbon, and five valence electrons per nitrogen.[8,9] The pseudopotentials were constructed using the generalized gradient approximation (GGA) of Perdew, Burke, and Ernzerhof (PBE).[10] For relaxations of supercells and for phonon calculations, we also used a DFT+U approach, where U is the Hubbard on-site potential.[11-13] A value of 4.2 eV for U was obtained using a linear response method.[14] For other calculations we employed a hybrid functional (Heyd-Scuseria-Ernzerhof, HSE)[15] with the standard range separation parameter $\omega = 0.2$. During our testing, we found that this hybrid approach was critical to maintain a finite band gap for $V(TCNE)_2$ (which is known to be semiconducting); the GGA, with or without a DFT+U approach, often underestimates band gaps.

Our own relaxed geometry of pure $V(TCNE)_2$ is consistent with previous work of De Fusco et al.[16] The $V(TCNE)_2$ cell has a triclinic structure and consists of one vanadium, 12 carbons and eight nitrogens. For the calculations involving adsorbed gas molecules, we inserted the gas molecules, oriented at various angles, into the open spaces within the calculated $V(TCNE)_2$ lattice between apical TCNE ligands, allowed the new structure to relax, and evaluated the formation energies. For most of our self-consistent calculations, we used 400 eV for the plane-wave energy cutoff, and a Γ-centered 3×3×3 k-mesh sampling. The relaxation calculations used an energy cut-toff of 530 eV and a Γ-centered 2×2×2 k-mesh sampling instead.

Shown in Figure S23 are the partial density of states of $V(TCNE)_2$ before (a) and after doping with (b) 0.25 and (c) 0.5 equivalents of ethylene molecules. The broad features of the valence band remain unchanged after the addition of ethylene. The conduction band is similarly mostly unaffected, although the presence of the ethylene appears to increase the effective band gap slightly, by about 0.3 eV. A small density of states associated with the ethylene p states is visible around 1.7 eV below the chemical potential. The small density of states associated with these ethylene p states suggests that this emerges from a very weak hybridization with the π* orbitals of TCNE; most of the ethylene density of states (occupied or unoccupied) is very far away from the chemical potential.

1.6 Surface area calculations

The surface area of $V(TCNE)_2$ was calculated using the open-source software Zeo++ version 0.36[17] accessible through Lawrence Berkeley Laboratory (LBNL) and the crystal structure of $V(TCNE)_2$ was obtained as described above (Section 1.5 Computational details). The geometrical surface area was calculated to be approximately 900 m^2/g with the spherical probe radius of 1.42 Å , which is similar to the kinetic diameters for H_2 but much smaller than N_2 and Ar. When we used a larger probe size, the framework pores became inaccessible by the probe since the probe cannot penetrate the rigid and defect-free framework. Similarly, we also performed calculations using Materials Studio. In this case, we simply estimated the occupied and free volume of $V(TCNE)_2$. With the void fraction, we estimated the pore volume of $V(TCNE)_2$ and converted to

the Langmuir surface area, assuming the cross-sectional area of N_2 is 16.2 $Å^2$/molecule. Then, the maximum Langmuir surface area was found to be approximately 920 m^2/g. While the threshold value of the probe radius is different, both calculations using two different softwares yield similar results. Considering that the pore volume of the $V(TCNE)_2$ model structure is not large enough for multi-layer gas adsorption, we believe that the geometrical surface area and Langmuir surface area should be quite similar to each other. In other words, the estimated surface area (900-920 m^2/g) by both methods are very close. The actual amorphous $V(TCNE)_2$ sample most likely exhibits a weakly-defined and less rigid framework structure. Further, the rotational freedom around the κ^2-$TCNE^-$ may allow a facile insertion of gas molecules during the adsorption process. Thus, it is unsurprising that $V(TCNE)_2$ adsorbs gas molecules with kinetic diameter larger than 3.4 Å.

2. Supporting Tables

Table S1. Fitting parameters to the tri-site Langmuir-Freundlich equation (eq. 1 and 2) for low-pressure H_2 adsorption at 77 and 87 K.

**Parameter**	**Values**		
	**Site 1**	**Site 2**	**Site 3**
$n_{sat,I}$ **(mmol/g)**	4.59	0.79	8.26
E_i **(−kJ/mol)**	5.8	4.9	10.2
S_i **(−J/mol·K)**	51.9	150.1	141.2
v_i	0.83	1.06	0.66

Table S2. Summary of room-temperature electronic conductivity measurements.

**Sample**	**$V(TCNE)_2 \cdot 0.95CH_2Cl_2$**	**Activated $V(TCNE)_2$**	**Aged $V(TCNE)_2$**
**Contact Area (cm^2)**	0.055(1)	0.055(1)	0.055(1)
**Pellet Thickness (cm)**	0.078(2)	0.085(2)	0.135(2)
**Conductivity (S/cm)**	$1.2(1) \times 10^{-3}$	$1.1(1) \times 10^{-3}$	$9.2(2) \times 10^{-5}$

3. Supporting Figures

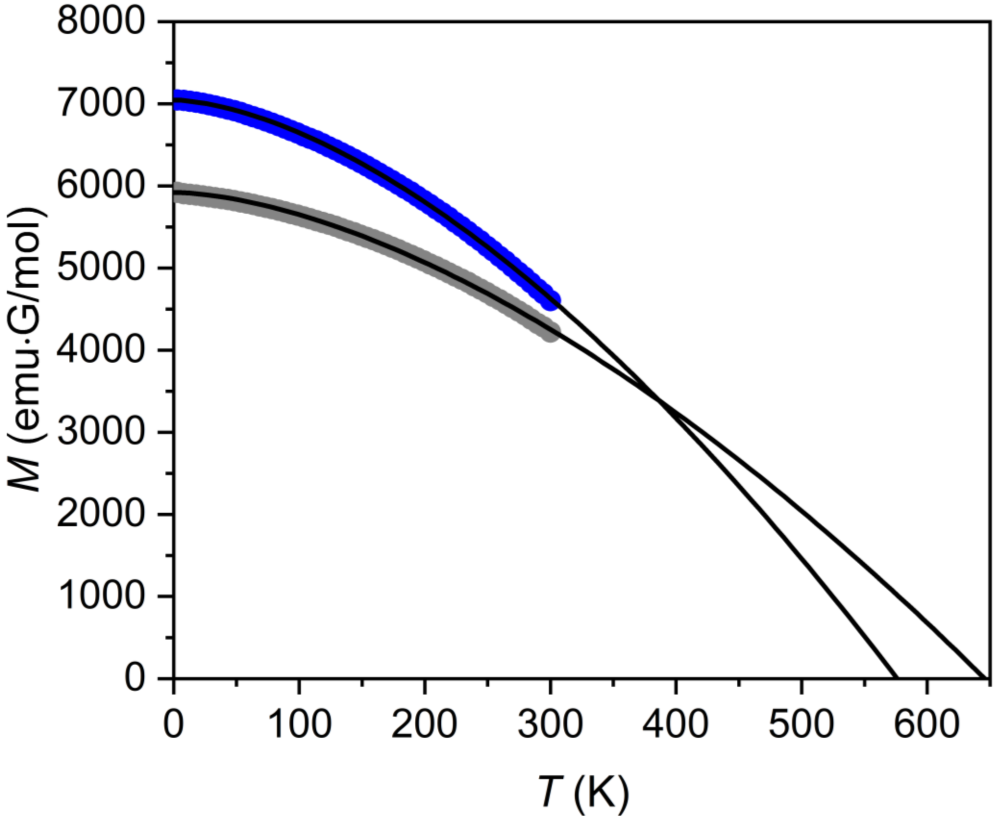

Figure S1. Variable-temperature field-cooled magnetic susceptibility data collected for $V(TCNE)_2{\cdot}0.95CH_2Cl_2$ (grey) and a sample $V(TCNE)_2$ after resolvation with CH_2Cl_2 (blue) as described in the Experimental Section of the main text. Data were collected using a dc field of 0.2 T. Black lines represent fits to the Bloch law.

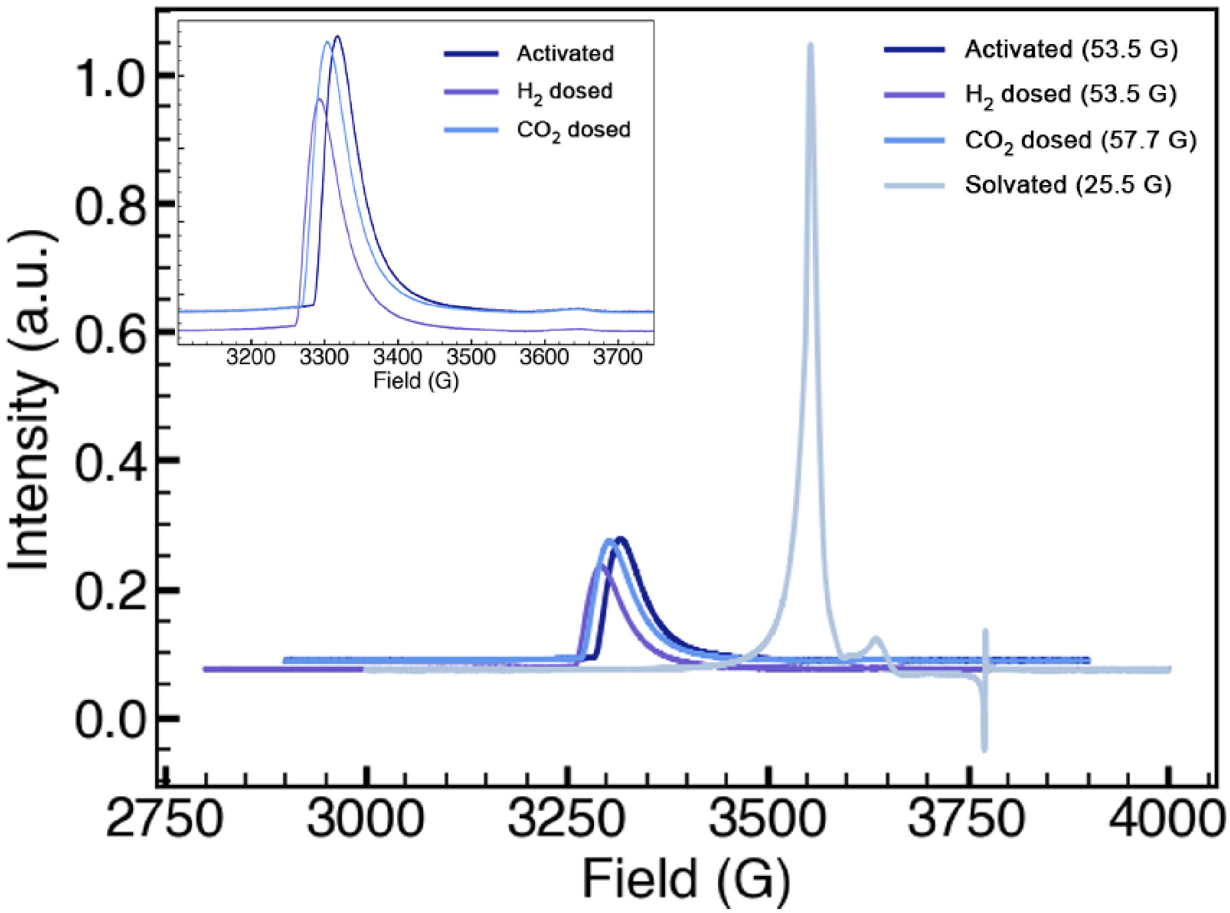

Figure S2. Ferromagnetic resonance (FMR) spectra for solvated $V(TCNE)_2 \cdot 0.95CH_2Cl_2$, activated $V(TCNE)_2$, and samples of activated $V(TCNE)_2$ dosed with CO_2 and H_2.

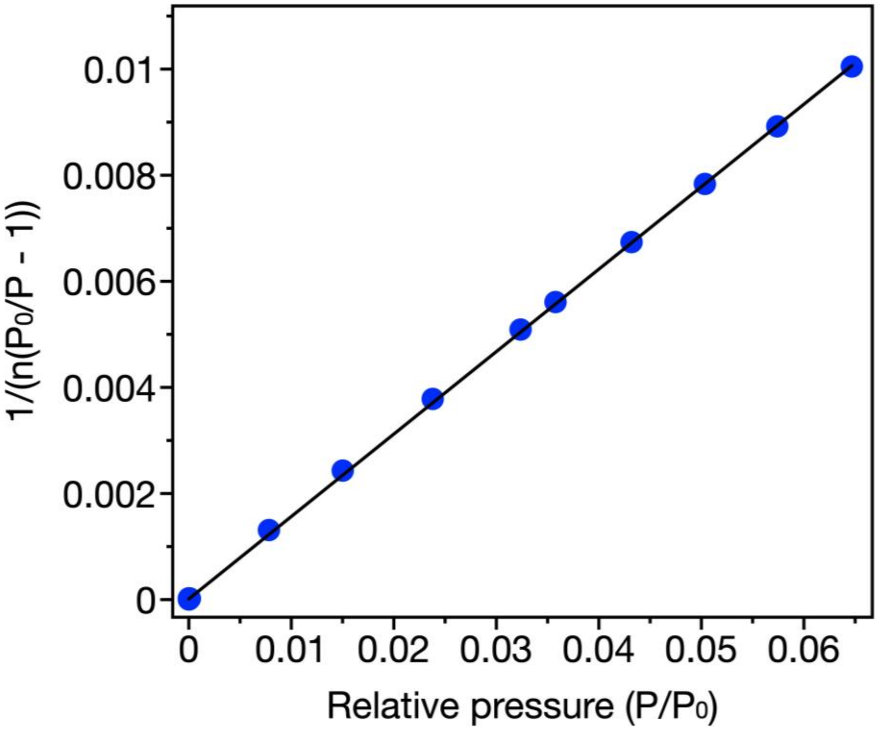

Figure S3. Calculation of BET surface area for activated $V(TCNE)_2$.

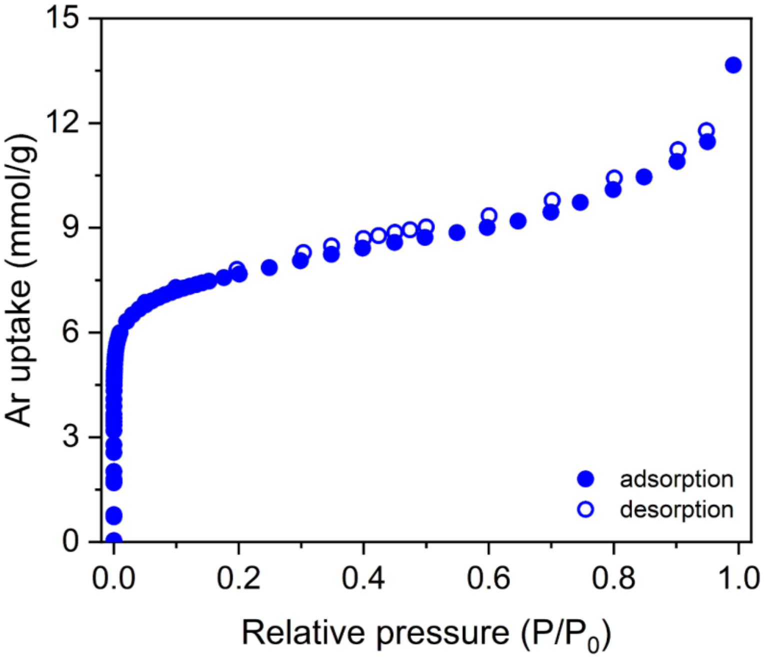

Figure S4. Argon adsorption data collected for $V(TCNE)_2$ at 87 K.

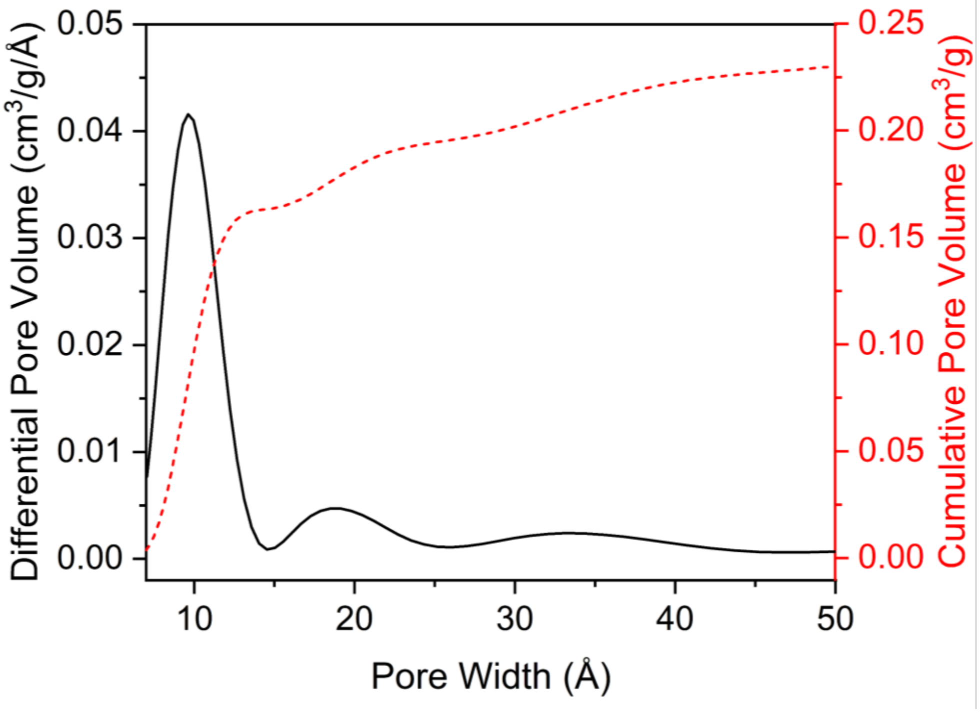

Figure S5. Pore size distribution analysis for activated $V(TCNE)_2$.

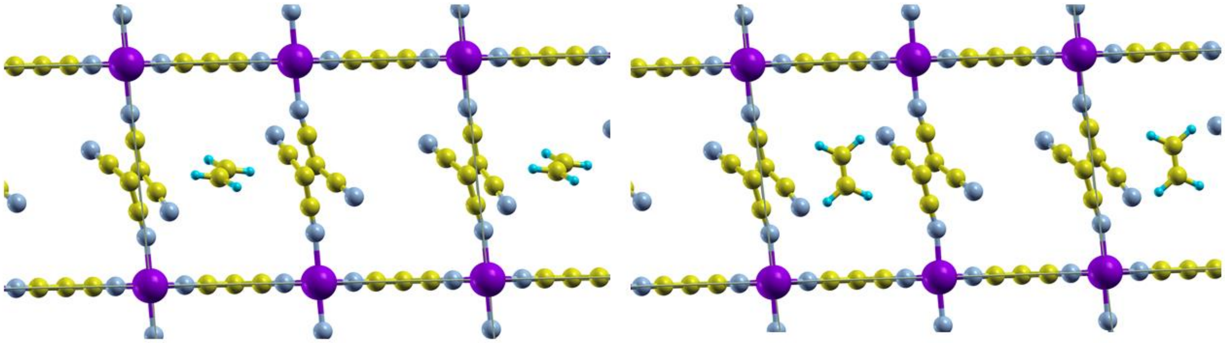

Figure S6. Two geometry-optimized structures of $V(TCNE)_2 \cdot 0.5C_2H_4$. Purple, yellow, grey, light blue spheres represent vanadium, carbon, nitrogen, and hydrogen atoms, respectively.

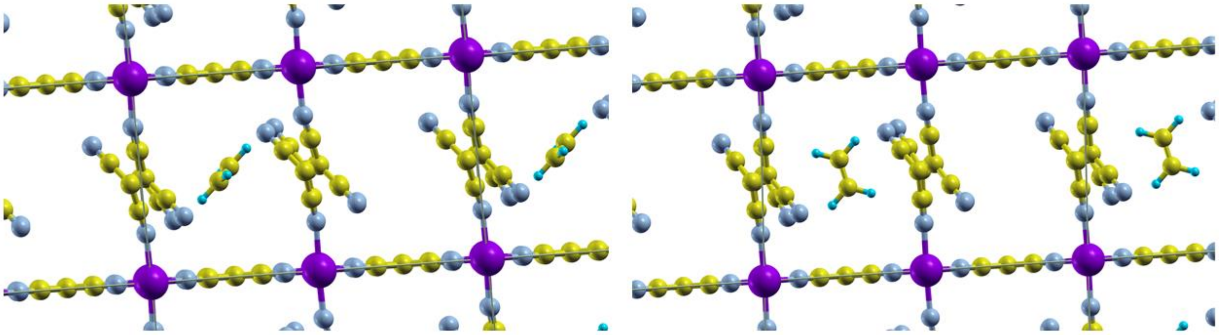

Figure S7. Two geometry-optimized structures of $V(TCNE)_2 \cdot 0.25C_2H_4$. Purple, yellow, grey, light blue spheres represent vanadium, carbon, nitrogen, and hydrogen atoms, respectively.

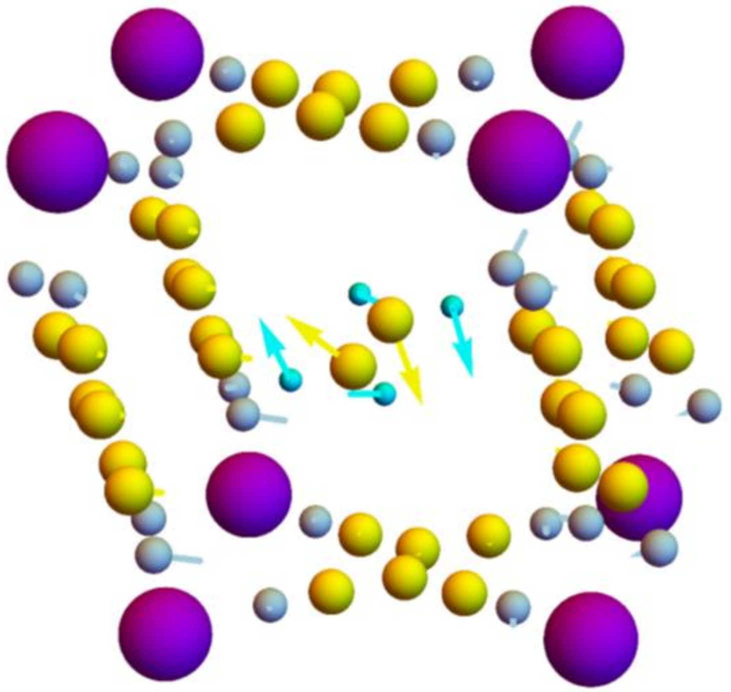

Figure S8. One vibration mode from phonon calculations of $V(TCNE)_2 \cdot 0.5C_2H_4$, illustrating rotation of an ethylene molecule in the pore. Purple, yellow, gray, light blue spheres represent vanadium, carbon, nitrogen, and hydrogen atoms, respectively. The sizes and directions of arrows indicate movements of connected atoms corresponding to this phonon mode.

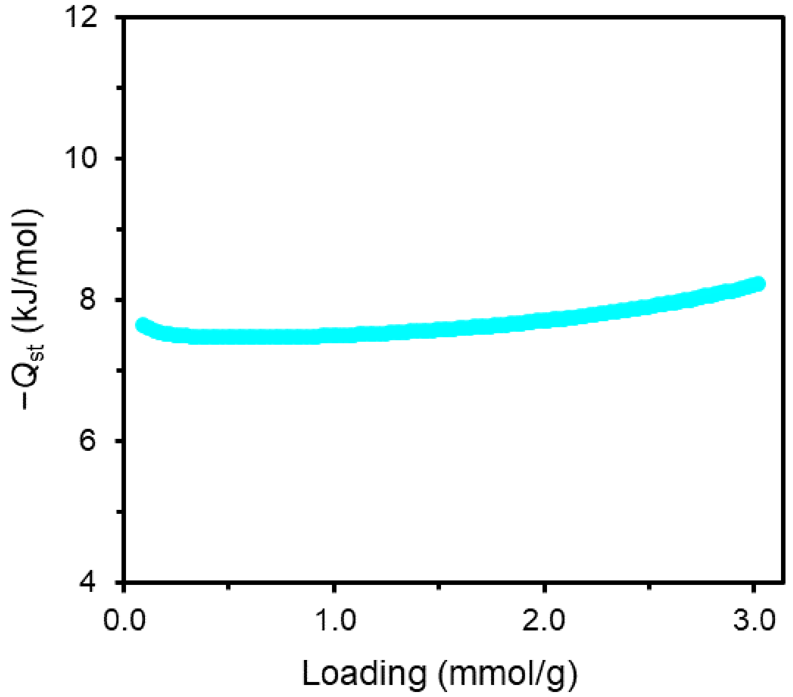

Figure S9. Isosteric heats of adsorption for H_2 as a function of loading in $V(TCNE)_2$, as determined from isotherms collected at 77 and 87 K.

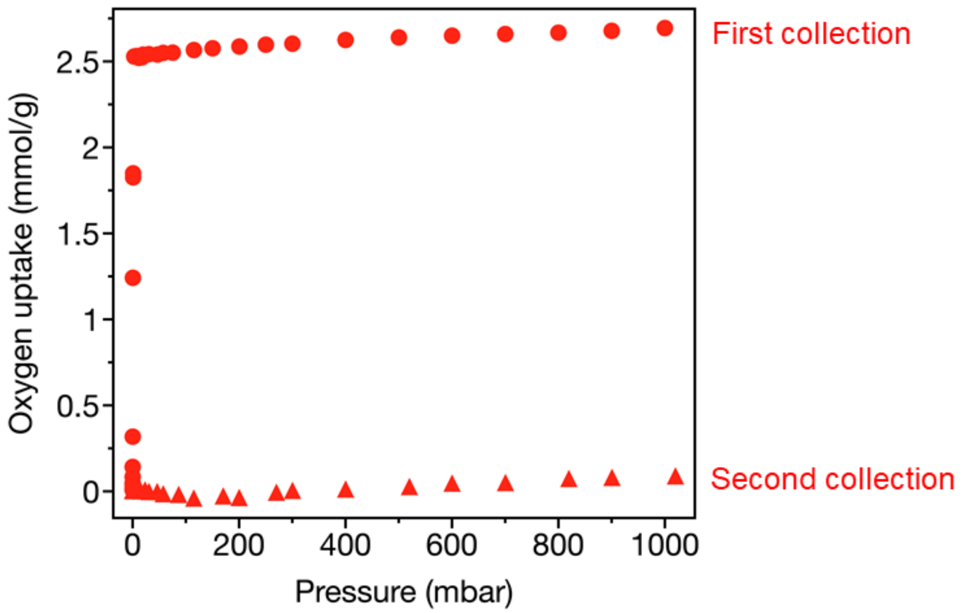

Figure S10. Oxygen adsorption data obtained at 298 K for activated $V(TCNE)_2$ (circles) and for the resulting O_2-saturated sample after it was exposed to high vacuum for approximately 3 hours at room temperature (triangles). Minimal uptake is observed following adsorption and vacuum exposure, corroborating that O_2 binding in $V(TCNE)_2$ under these conditions is not reversible.

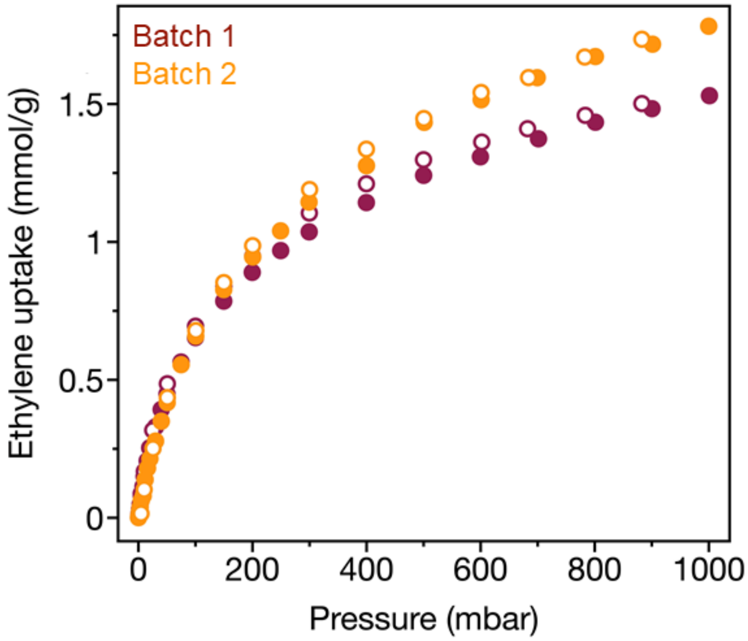

Figure S11. Ethylene adsorption data obtained at 298 K for two different batches of activated $V(TCNE)_2$ prepared using identical synthetic conditions.

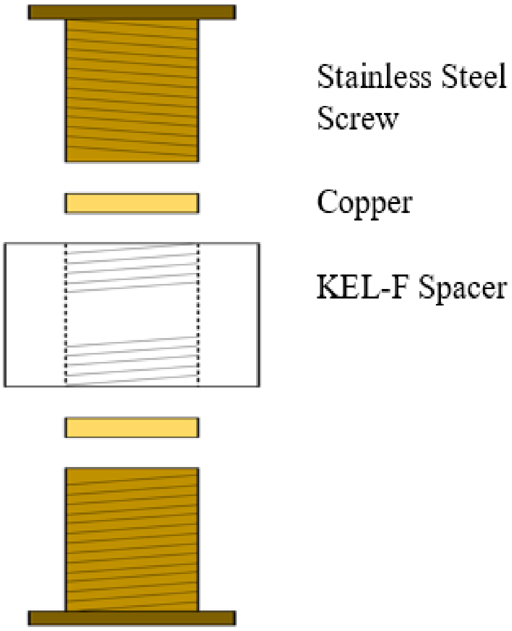

Figure S12. Custom-designed two contact pressed pellet screw cell.

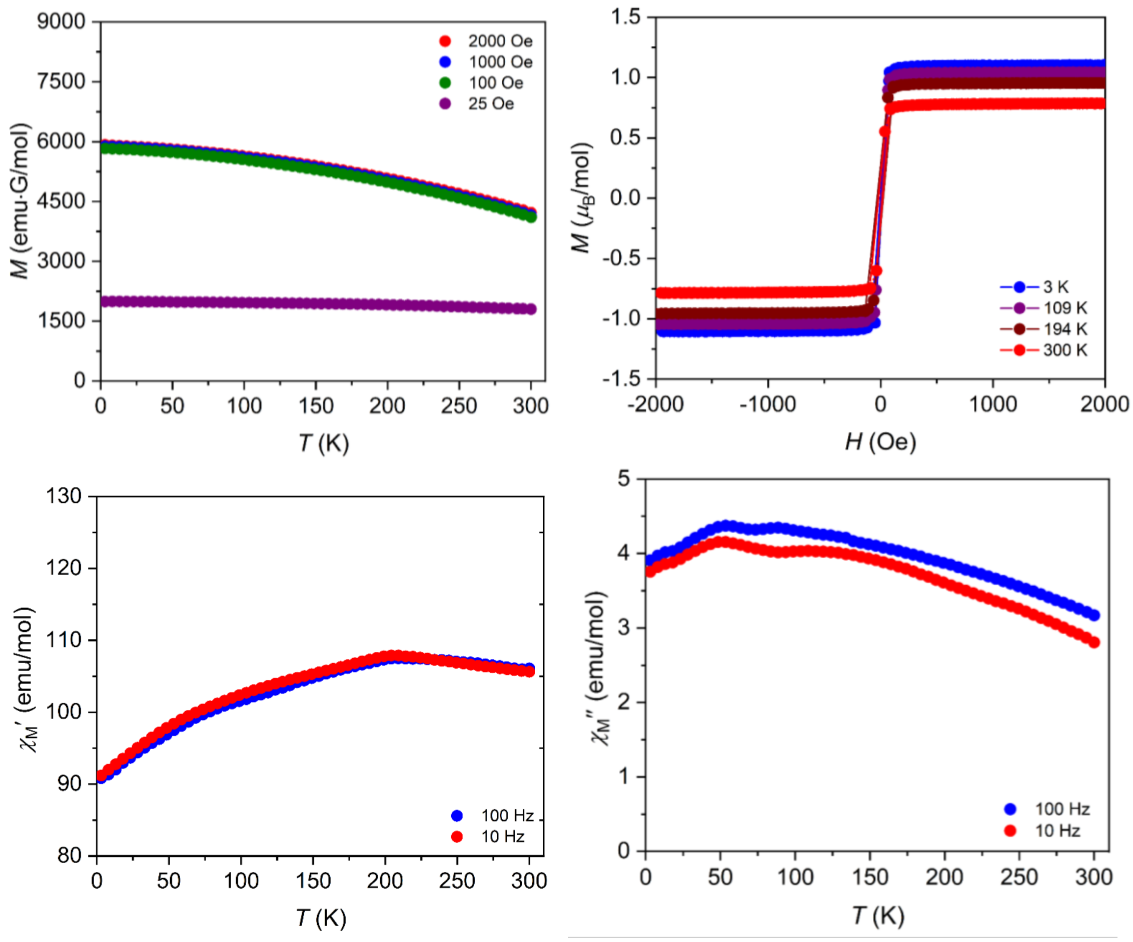

Figure S13. Magnetic characterization data for solvated $V(TCNE)_2{\cdot}0.95CH_2Cl_2$. (Top left) Variable-temperature field-cooled magnetic susceptibility data collected at dc magnetic fields of 25, 100, 1000, and 2000 Oe. (Top right) Variable-field magnetization data collected at selected temperatures. (Bottom) Variable-temperature in-phase (χ_M', left) and out-of-phase (χ_M'', right) ac magnetic susceptibility data collected with an oscillating magnetic field of 4 Oe at frequencies of 10 and 100 Hz under zero dc magnetic field.

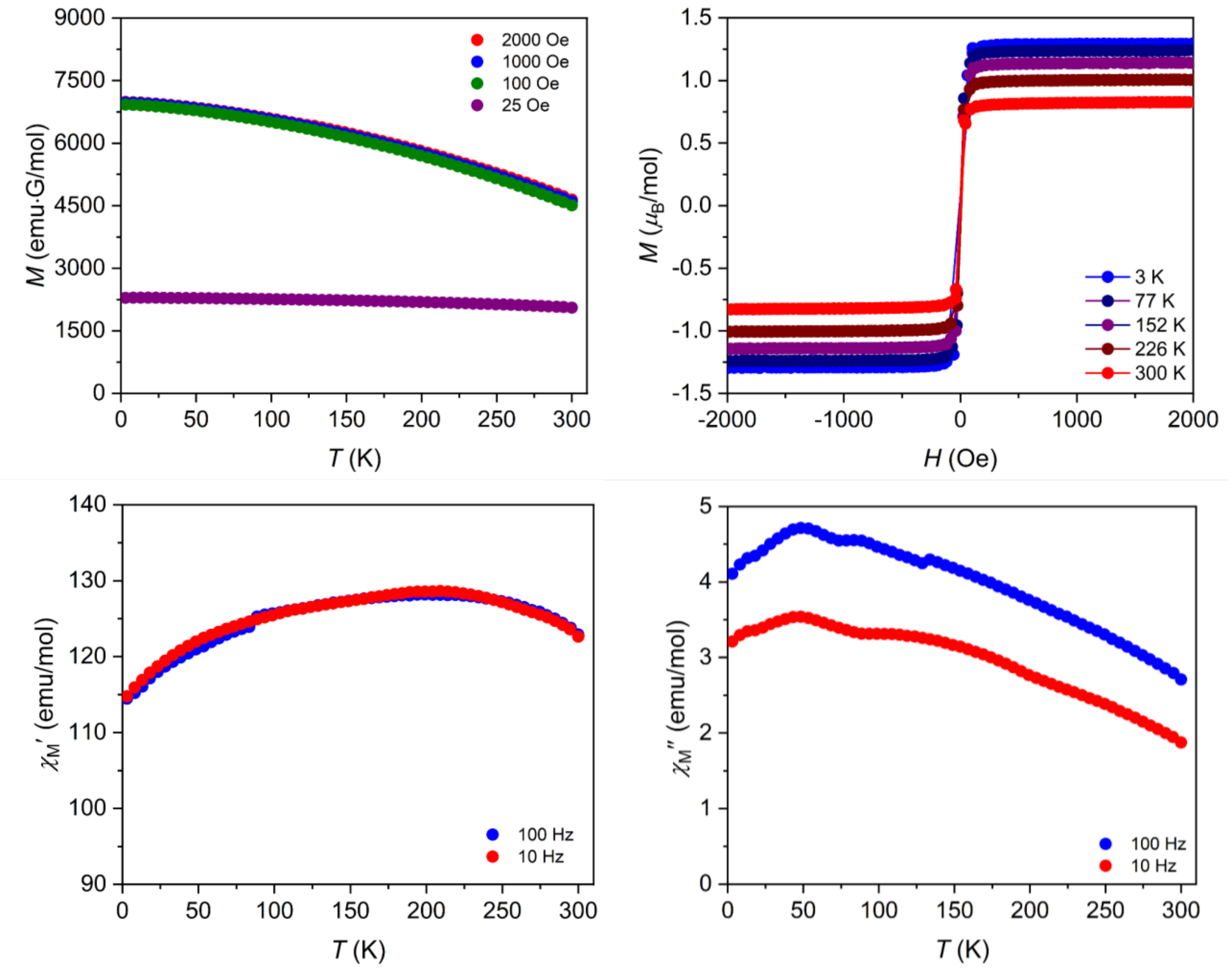

Figure S14. Magnetic characterization data for activated $V(TCNE)_2$. (Top left) Variable-temperature field-cooled magnetic susceptibility data collected at dc magnetic fields of 25, 100, 1000, and 2000 Oe. (Top right) Variable-field magnetization data collected at selected temperatures. (Bottom) Variable-temperature in-phase (χ_M', left) and out-of-phase (χ_M'', right) ac magnetic susceptibility data collected with an oscillating magnetic field of 4 Oe at frequencies of 10 and 100 Hz under zero dc magnetic field.

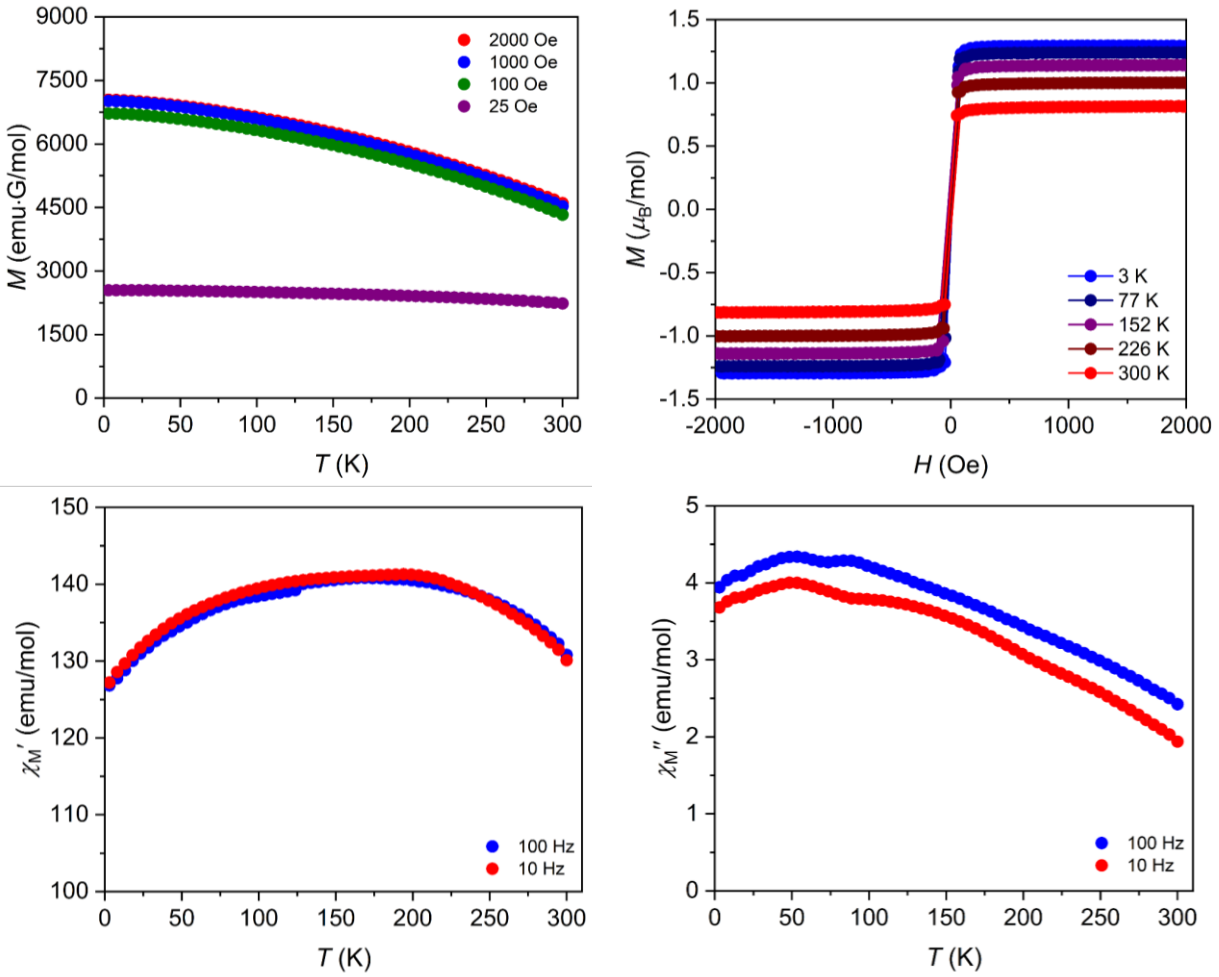

Figure S15. Magnetic properties of $V(TCNE)_2$ resolvated with dichloromethane. (Top left) Variable-temperature field-cooled magnetic susceptibility data collected at dc magnetic fields of 25, 100, 1000, and 2000 Oe. (Top right) Variable-field magnetization data collected at selected temperatures. (Bottom) Variable-temperature in-phase (χ_M', left) and out-of-phase (χ_M'', right) ac magnetic susceptibility data collected with an oscillating magnetic field of 4 Oe at frequencies of 10 and 100 Hz and under zero dc magnetic field.

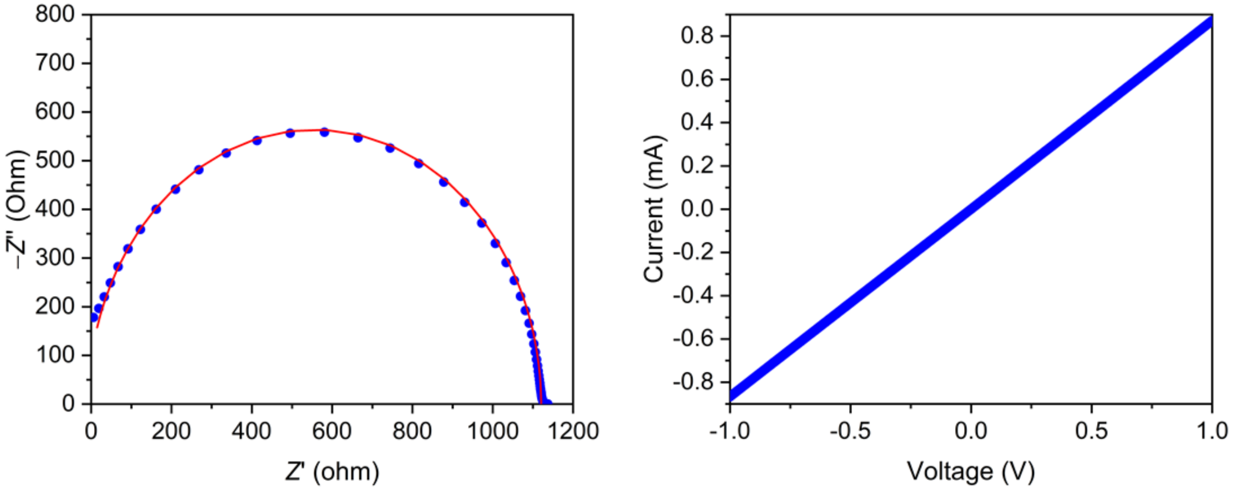

Figure S16. Electronic conductivity data obtained for $V(TCNE)_2{\cdot}0.95CH_2Cl_2$ using electrochemical impedance spectroscopy (left) and *I-V* measurements (right). The fit to the data at left is shown in red.

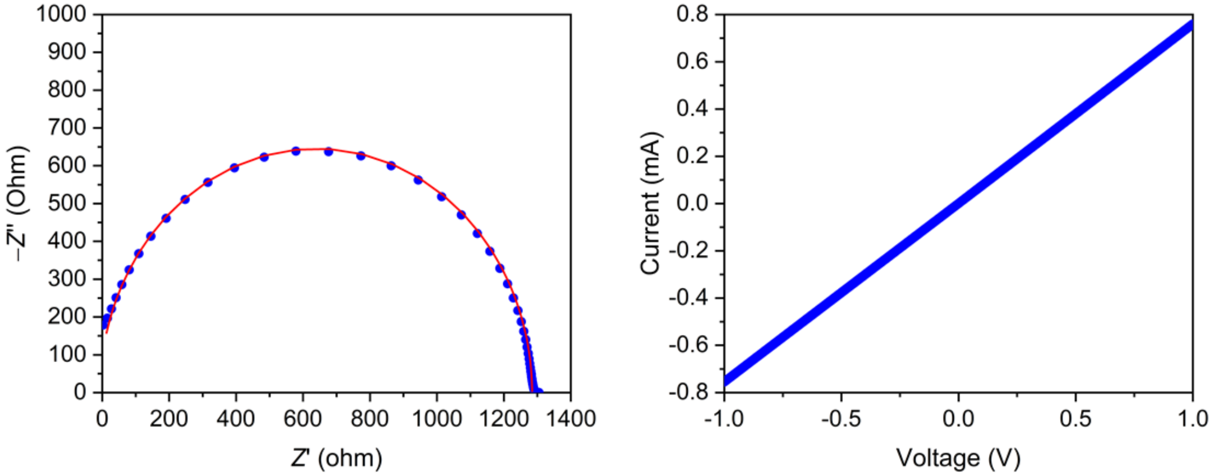

Figure S17. Electronic conductivity data obtained for activated $V(TCNE)_2$ using electrochemical impedance spectroscopy (left) and *I-V* measurements (right). The fit to the data at left is shown in red.

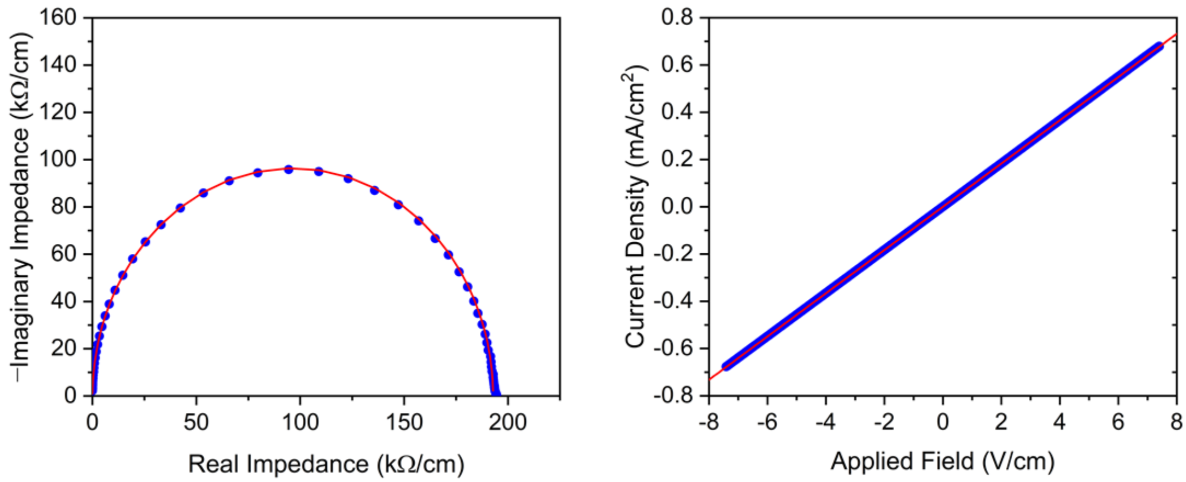

Figure S18. Electronic conductivity data obtained for an aged sample of $V(TCNE)_2$ (stored under Ar at 295 K for one month) using electrochemical impedance spectroscopy (left) and *I-V* measurements (right). Fits to the data are shown in red.

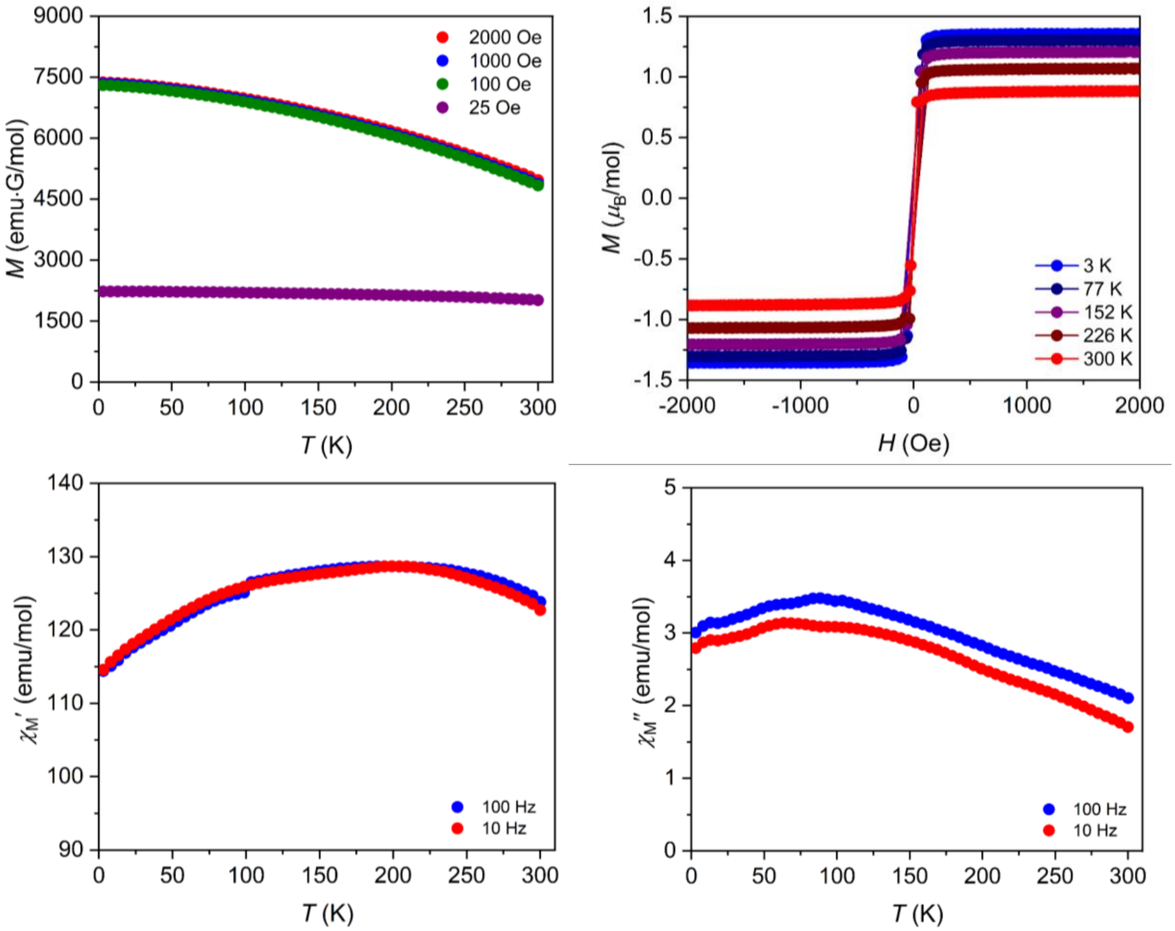

Figure S19. Magnetic characterization data obtained for activated $V(TCNE)_2$ dosed with CO_2. (Top left) Variable-temperature field-cooled magnetic susceptibility data collected at dc magnetic fields of 25, 100, 1000, and 2000 Oe. (Top right) Variable-field magnetization data collected at selected temperatures. (Bottom) Variable-temperature in-phase (χ_M', right) and out-of-phase (χ_M'', left) ac magnetic susceptibility data collected with an oscillating magnetic field of 4 Oe at frequencies of 10 and 100 Hz under zero dc magnetic field.

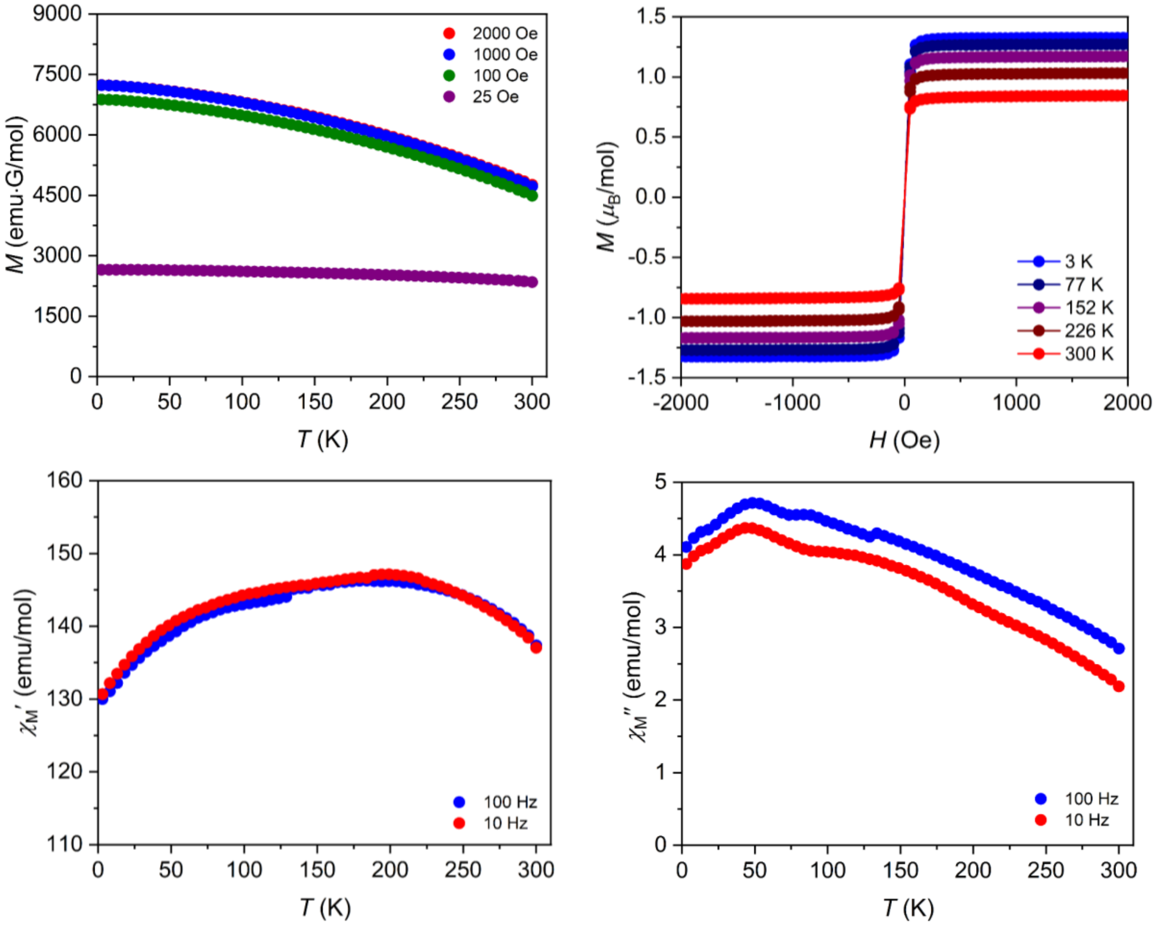

Figure S20. Magnetic characterization data obtained for activated $V(TCNE)_2$ dosed with H_2. (Top left) Variable-temperature field-cooled magnetic susceptibility data collected at dc magnetic fields of 25, 100, 1000, and 2000 Oe. (Top right) Variable-field magnetization data collected at selected temperatures. (Bottom) Variable-temperature in-phase (χ_M', left) and out-of-phase (χ_M'', right) ac magnetic susceptibility data collected with an oscillating magnetic field of 4 Oe at frequencies of 10 and 100 Hz under zero dc magnetic field.

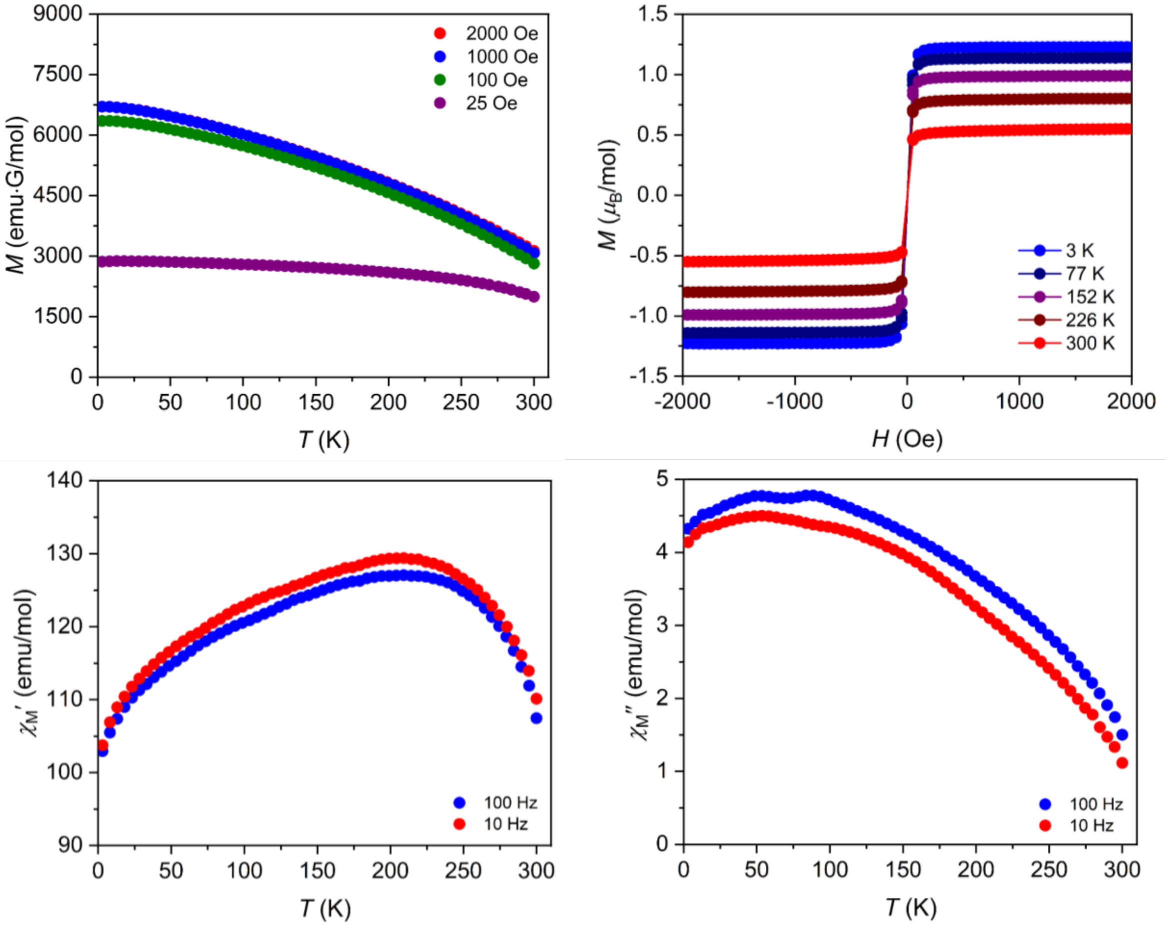

Figure S21. Magnetic characterization data obtained for activated $V(TCNE)_2$ dosed with ethylene. (Top left) Variable-temperature field-cooled magnetic susceptibility data collected at dc magnetic fields of 25, 100, 1000, and 2000 Oe. (Top right) Variable-field magnetization data collected at selected temperatures. (Bottom) Variable-temperature in-phase (χ_M', left) and out-of-phase (χ_M'', right) ac magnetic susceptibility data collected with an oscillating magnetic field of 4 Oe at frequencies of 10 and 100 Hz under zero dc magnetic field.

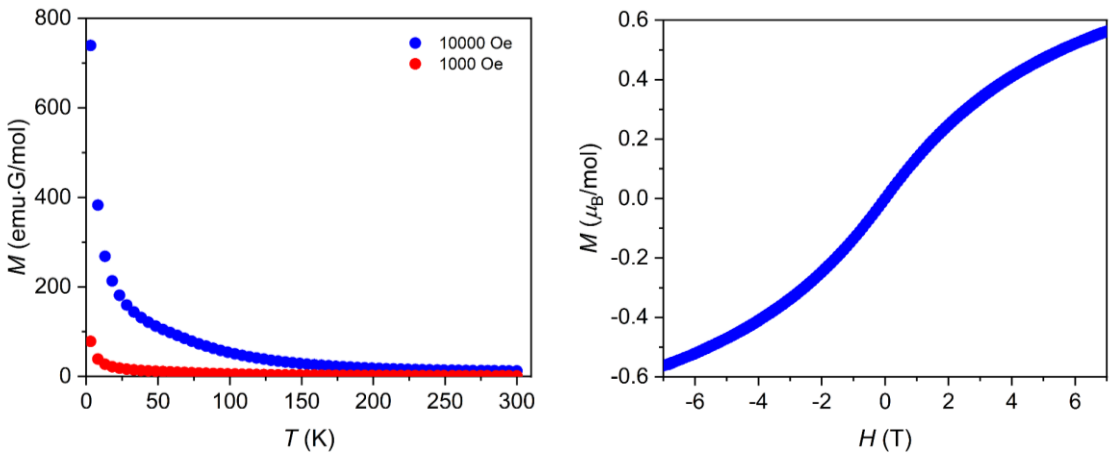

Figure S22. Magnetic characterization data obtained for activated $V(TCNE)_2$ dosed with O_2 at 300 K. (Left) Variable-temperature field-cooled magnetic susceptibility data collected at dc magnetic fields of 1000 and 10000 Oe. (Right) Variable-field magnetization data collected at 3 K.

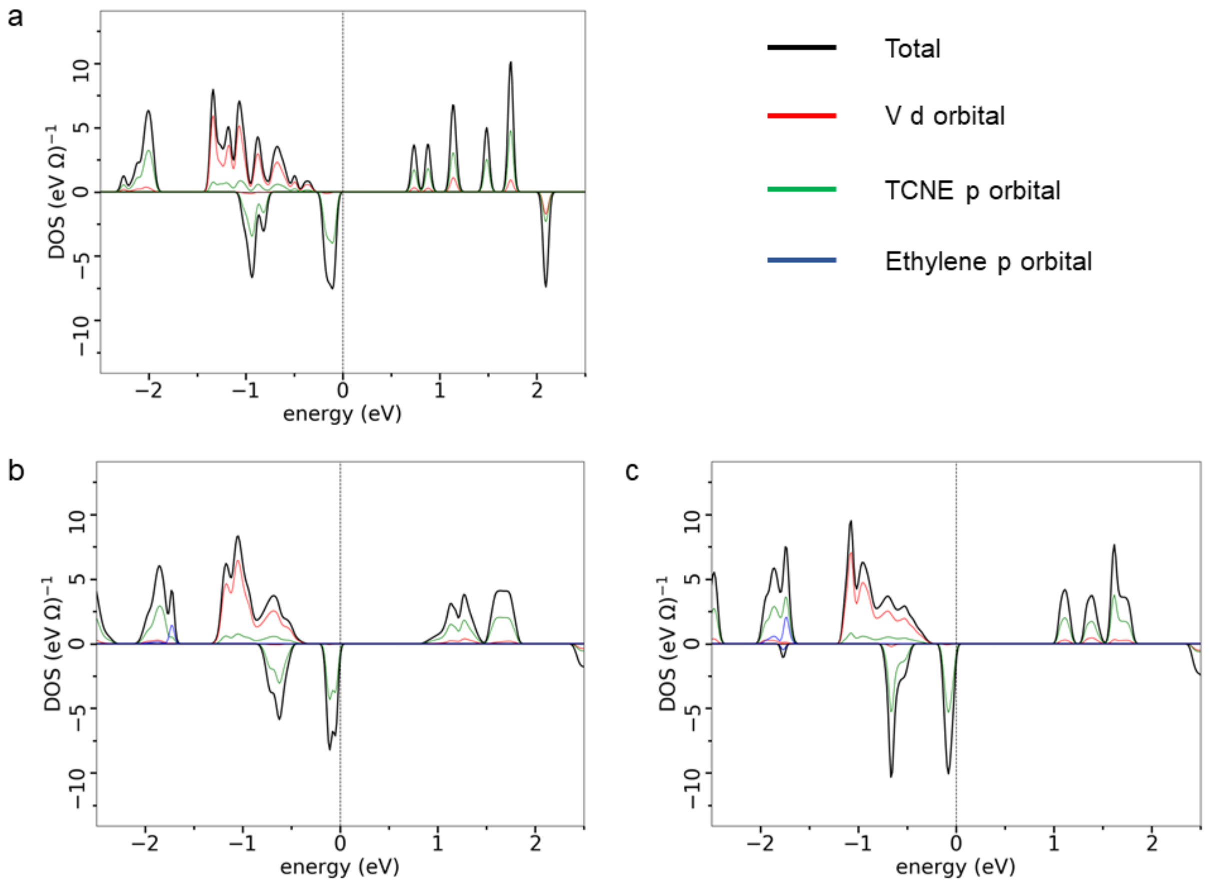

Figure S23. Partial density of states of $V(TCNE)_2$ (a) and $V(TCNE)_2$ doped with (b) 0.25 and (c) 0.5 equivalents of ethylene, as obtained from electronic structure calculations. The spin-polarized d states of the vanadium ion are shown in red, and the oppositely-polarized p states of the TCNE molecules are shown in green. A small density of states associated with the ethylene p states is visible around 1.7 eV below the chemical potential, and is indicated in blue.

4. References

(1) Brinckerhoff, W. B.; Zhang, J.; Miller, J. S.; Epstein, A. J. Magnetization of High-T_C Molecule-Based Magnet V/TCNE/CH_2Cl_2. *J. Mol. Cryst. Liq. Cryst.* **1995**, *272*, 195-205.

(2) Morin, B. G.; Zhou, P.; Hahm, C.; Epstein, A. J.; Miller, J. S. Complex ac susceptibility studies of the disordered molecular based magnets V(TCNE)$_x$: Role of spinless solvent. *J. Appl. Phys.* **1993**, *73*, 5648-5650.

(3) Pokhodnya, K. I.; Pejakovic, D.; Epstein, A. J.; Miller, J. S. Effect of solvent on the magnetic properties of the high-temperature V[TCNE]$_x$ molecule-based magnet. *Phys. Rev. B* **2001**, *63*, 174408.

(4) Morin, B. G.; Hahm, C.; Miller, J. S.; Epstein, A. J. Molecular magnets V(tetracyanoethylene)$_x$·y(solvent): Applications to magnetic shielding. *J. Appl. Phys.* **1994**, *75*, 5782-5784.

(5) Kresse, G.; Hafner, J. *Ab initio* molecular dynamics for liquid metals. *Phys. Rev. B* **1993**, *47*, 558-561.

(6) Kresse, G.; Furthmüller, J. Efficiency of ab-initio total energy calculations for metals and semiconductors using a plane-wave basis set. *Comput. Mat. Sci.* **1996**, *6*, 15-50.

(7) Kresse, G.; Furthmüller, J. Efficient iterative schemes for ab initio total-energy calculations using a plane-wave basis set. *Phys. Rev. B* **1996**, *54*, 11169-11186.

(8) Kresse, G.; Hafner, J. Norm-coserving and ultrasoft pseudopotentials for first-row and transition elements. *J. Phys. Condens. Matt.* **1994**, *6*, 8245-8257.

(9) Kresse, G.; Joubert, D. From ultrasoft pseudopotentials to the projector augmented-wave method. *Phys. Rev. B* **1999**, *59*, 1758-1775.

(10) Perdew, J. P.; Burke, K; Ernzerhof, M. Generalized Gradient Approximation Made Simple. *Phys. Rev. Lett.* **1996**, *77*, 3865-3868.

(11) Anisimov, V.; Zaanen, J.; Andersen, O. Band theory and Mott insulators: Hubbard *U* instead of Stoner *I*. *Phys. Rev. B* **1991**, *44*, 943-954.

(12) Anisimov, V.; Solovyev, I.; Korotin, M.; Czyzyk, M.; Sawatzky, G. Density-functional theory and NiO photoemission spectra. *Phys. Rev. B* **1993**, *48*, 16929-16934.

(13) Solovyev, I.; Dederichs, P.; Anisimov, V. Corrected atomic limit in the local-density approximation and the electronic structure of *d* impurities in Rb. *Phys. Rev. B* **1994**, *50*, 16861-16871.

(14) Kulik, H. J.; Cococcioni, M.; Scherlis, D. A.; Marzari, N. Density Functional Theory in Transition-Metal Chemistry: A Self-Consistent Hubbard *U* Approach. *Phys. Rev. Lett.* **2006**, *97*, 103001.

(15) Heyd, J.; Scuseria, G. E.; Ernzerhof, M. Hybrid functionals based on a screened Coulomb potential. *J. Chem. Phys.* **2003**, *118*, 8207-8215.

(16) De Fusco, G. C.; Pisani, L.; Montanari, B.; Harrison, N. M. Density functional study of the magnetic coupling in V(TCNE)$_2$. *Phys. Rev. B* **2009**, *79*, 085201.

(17) Willems, T. F.; Rycroft, C. H.; Kazi, M.; Meza, J. C.; Haranczyk, M. Algorithms and tools for high-throughput geometry-based analysis of crystalline porous materials. *Micro. Meso. Mater.* **2012**, *149*, 134-141.
